# Identification of Bacterial Blight Resistance Loci in Rice (*Oryza sativa* L.) against Diverse *Xoo* Thai Strains by Genome-Wide Association Study

**DOI:** 10.3390/plants10030518

**Published:** 2021-03-10

**Authors:** Siriporn Korinsak, Clive T. Darwell, Samart Wanchana, Lawan Praphaisal, Siripar Korinsak, Burin Thunnom, Sujin Patarapuwadol, Theerayut Toojinda

**Affiliations:** 1 National Center for Genetic Engineering and Biotechnology (BIOTEC), 113 Thailand Science Park, Pahonyothin Road, Khlong Nueng, Khlong Luang, Pathum Thani 12120, Thailand; siripornkorinsak@gmail.com (S.K.); cliveterence.dar@biotec.or.th (C.T.D.); samart.wan@biotec.or.th (S.W.); yingyuy18@gmail.com (L.P.); siripar.skk@gmail.com (S.K.); burinthnm@gmail.com (B.T.); 2 Department of Plant Pathology, Faculty of Agriculture at Kamphaeng Saen, Kasetsart University, Kamphaeng Saen Campus, Nakhon Pathom 73140, Thailand; agrsujp@ku.ac.th

**Keywords:** bacterial leaf blight, rice, GWAS, *Xanthomonas oryzae* pv. *oryzae*

## Abstract

Bacterial leaf blight (BLB) is a serious disease affecting global rice agriculture caused by *Xanthomonas oryzae* pv. *oryzae* (*Xoo*). Most resistant rice lines are dependent on single genes that are vulnerable to *resistance breakdown* caused by pathogen mutation. Here we describe a genome-wide association study of 222 predominantly Thai rice accessions assayed by phenotypic screening against 20 *Xoo* isolates. Loci corresponding to BLB resistance were detected using >142,000 SNPs. We identified 147 genes according to employed significance thresholds across chromosomes 1–6, 8, 9 and 11. Moreover, 127 of identified genes are located on chromosomal regions outside estimated Linkage Disequilibrium influences of known resistance genes, potentially indicating novel BLB resistance markers. However, significantly associated SNPs only occurred across a maximum of six *Xoo* isolates indicating that the development of broad-spectrum *Xoo* strain varieties may prove challenging. Analyses indicated a range of gene functions likely underpinning BLB resistance. In accordance with previous studies of accession panels focusing on *indica* varieties, our germplasm displays large numbers of SNPs associated with resistance. Despite encouraging data suggesting that many loci contribute to resistance, our findings corroborate previous inferences that multi-strain resistant varieties may not be easily realised in breeding programs without resorting to multi-locus strategies.

## 1. Introduction

Bacterial leaf blight (BLB), caused by *Xanthomonas oryzae* pv. *oryzae* (*Xoo*), is a serious crop disease causing major losses to rice production around the world [1]. BLB has been estimated to cause 20–80% of rice yield loss [2,3,4,5]. In recent decades, increases in BLB outbreaks have been recorded and often attributed to global temperature rises linked to ongoing anthropogenic climate change [6,7,8]. Additionally, increases in genetic diversity of *Xoo*, and emergence of new races are regularly reported in intensive rice production areas where susceptible rice varieties are often used.

The use of diverse rice varieties [9], misapplication of chemicals [10] and natural mutations of the pathogen [11], have been suggested as the agronomic drivers exacerbating the emergence of novel *Xoo* races and assisting their host shifts across rice varieties and to new geographic localities. In Thailand, outbreaks have been regularly reported since 1957 [12,13,14] and high genetic diversity among *Xoo* isolates has been documented [12,13]. There are a growing number of reports on the genetic diversity of *Xoo* at both regional and national scales that likely result from practices of continuous mono-cropping, the deployment of rice cultivars with a narrow genetic base, and the anthropogenically mediated crossing of geographical barriers through seed allocation and germplasm exchange [9,12,15,16,17,18,19].

It is established that rice cultivars that rely on single major genes for resistance are more susceptible to suffer resistance breakdown by pathogen mutational dynamics than cultivars featuring multi-locus-based resistance phenotypes [20]. Thus, breeding programs that target the development of multi-locus resistance varieties should prove a more viable strategy to ensure long-term goals in sustainable rice production [21,22,23,24].

Numerous resistance (R) genes for BLB have been discovered during the last 50 years. At least 43 resistance genes (labelled with *R* prefixes) have been identified and characterized from various rice accessions including its wild relatives [25,26,27,28,29,30,31,32,33,34,35,36]. Of these, 16 genes reportedly function as recessive genes with the rest characterized as dominant [37]. Gene functionality is often shown to be influenced by genetic background and plant developmental stage, e.g., *Xa1, Xa3, Xa21, xa25, Xa26* [38]. Currently, nine *R* genes (*Xa1, Xa3/Xa26, xa5, Xa10, xa13, Xa23, xa25, Xa27, Xa21*) have been cloned [35,39,40,41,42,43,44,45,46], and are thus associated with defined chromosomal locations relative to rice reference genomes. The cloned *R* genes encode various types of protein such as a cytoplasmic domain containing a serine-threonine kinase, a transmembrane domain, an extracellular domain with leucine rich repeat (LRR) receptor kinase like proteins, NB-LRR protein, the gamma subunit of transcription factor IIA (TFIIAγ), a plasma membrane protein of the MtN3/saliva family, and an unknown protein [38,39,40,42,43,45,47,48]. Although the identification of *R* genes is increasing rapidly, the development of race/isolate specific or broad-spectrum resistance against *Xoo* remains elusive.

Recently, current advances in high-density molecular marker platforms and the application of genetic resources with assorted diversity panels has enabled the accurate identification of genomic locations and candidate genes for traits of interest through genome-wide association studies (GWAS) and allele mining. GWAS has been widely applied to identify genes of interest in a number of crop species, such as rice, maize, sugarcane, cotton, wheat, barley, potato and soybean [49,50,51,52]. GWAS methods permit the identification of novel alleles that are likely to be useful in crop improvement programs [53]. In rice, GWAS has been used to identify DNA markers associated with traits for grain quality, yield (and its correlates), stigma and spikelet characteristics, eating and cooking qualities, and diseases resistance such as for sheath blight, blast and BLB [24,25,54,55,56,57,58,59]. GWAS studies on BLB have identified loci associated with cloned and fine-mapped genes [25,54] while analysis on *Xoo* strains originating from the Philippines yielded SNPs that did not overlap with any known resistance loci [24].

Although several BLB resistance genes have been discovered, their effectiveness depends upon pathogenic variability and the rate at which mutational adaptation overcomes resistance phenotypes. Identification of genes that confer resistance against different *Xoo* races is crucial in developing broad-scale resistant rice varieties. The discovery of novel resistance alleles will facilitate the development of new resistant cultivars. Here we aim to identify resistance loci and SNP markers in 222 accessions of rice germplasm that have been previously subject to whole genome sequencing (WGS) analysis by performing phenotypic assay of BLB against 20 *Xoo* isolates. Our work is intended to contribute to future BLB breeding programs both in Thailand and the rest of the world.

## 2. Results

### 2.1. Phenotypic Screening

**Figure 1:**
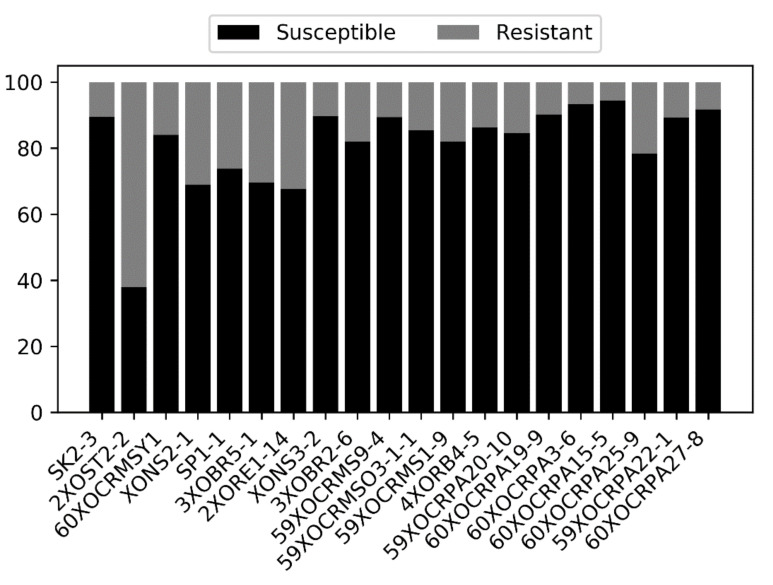
Percentage of susceptible versus resistant lines across 20 focal *Xoo* isolates (x-axis). Most rice accessions are predominantly susceptible to all but one *Xoo* isolate.

Seedlings from our 222-accession (lines) panel were inoculated with 20 *Xoo* (bacterial leaf blight; BLB) isolates. From these, 19 isolates yielded a higher proportion of BLB susceptibility versus resistance across the 222 accessions (Table S1; R = 5.6–32.4%; S = 67.6–94.4%). Only one isolate, 2XOST2-2 yielded a greater number of resistant versus susceptible lines (R = 62.1%: S = 37.9%) (Figure 1).

**Figure 2:**
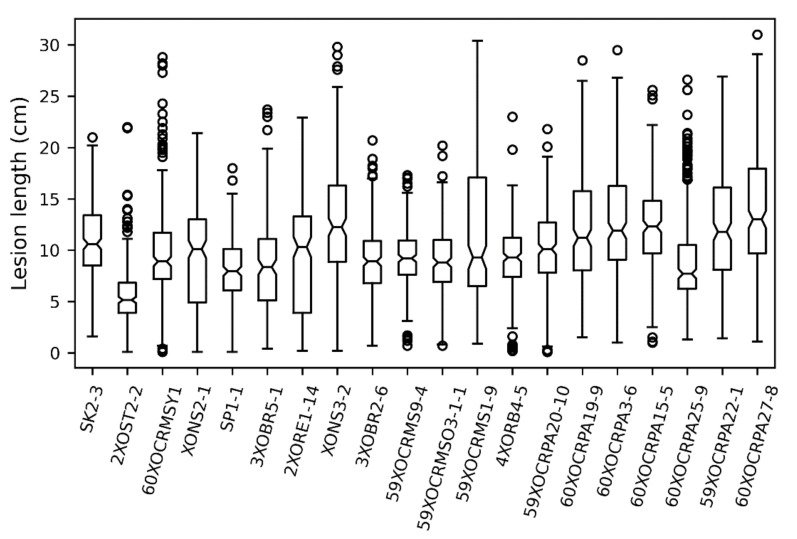
Leaf lesion length scores for BLB damage for rice panel cultivars across 20 focal *Xoo* isolates. Most *Xoo* isolates cause serious leaf damage.

Leaf lesion length scores, resulting from BLB infection, showed marked variation according to *Xoo* strain across our 222-sample panel with mean values ranging from 5.79 (strain 2XOST2_2) to 13.93 cm (strain 60XOCRPA27_8) (Figure 2). Additionally, large error bars imply that lesion length varies substantially according to assayed rice variety.

### 2.2. Population Structure of Rice Accessions

**Figure 3:**
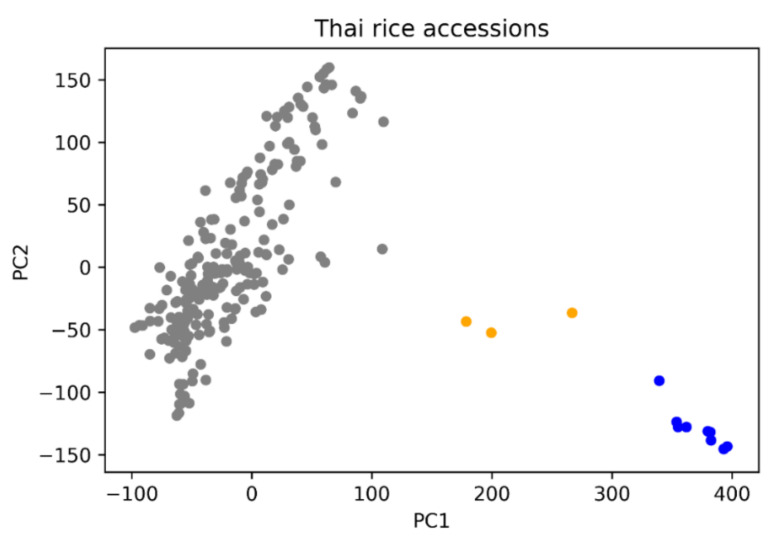
Principal component analysis of 222 accessions from our rice panel. Grey colour (PC1 <= 100) are *indica* varieties with a handful of *japonica* (blue) and *aus*/*aro* varieties (orange).

After filtering our genomic data, we identified 142,362 high-quality SNPs across 222 rice accessions. Principal component analysis (PCA) indicates that samples comprise of three genomic clusters (Figure 3). PCA when combined with typed reference samples from the IRRI database (Figure S1), indicated that most Thai samples are predominantly of *indica* origin. Additionally, nine accessions are *japonica* origin (blue), while three accessions also cluster with IRRI-typed *aus* samples (orange).

**Figure 4:**
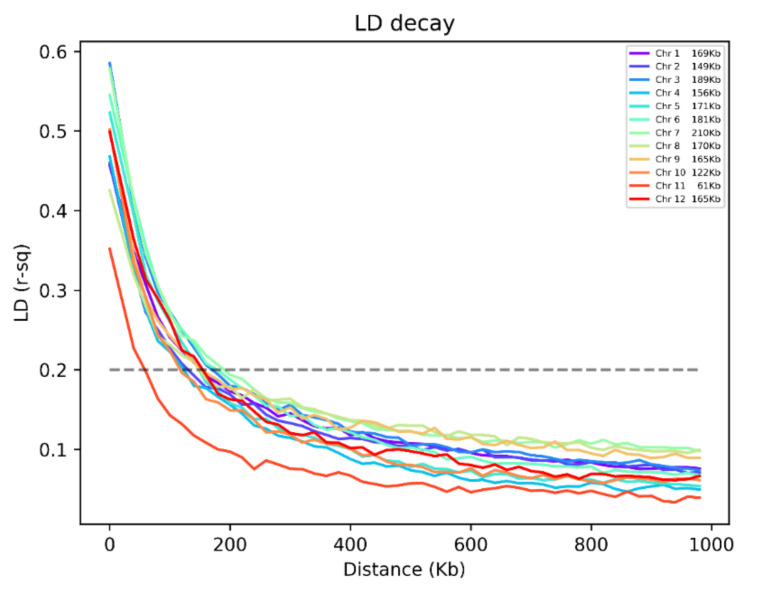
Linkage disequilibrium decay across 12 chromosomes in 222 *O. sativa* accessions. Mean LD decay ranges between 61–210 Kb.

We then conducted linkage disequilibrium (LD) decay analyses to evaluate chromosomal signatures of recombination patterns (Figure 4). Mean LD decay values within intra-chromosomal distance bins drop below a threshold mean r^2^ value of 0.2 between 61–201 Kb across all 12 chromosomes. Signatures for all chromosomes appear similar except for chromosome 11 which has a markedly lower (61 Kb) threshold crossover point.

### 2.3. GWAS Analysis

**Figure 5:**
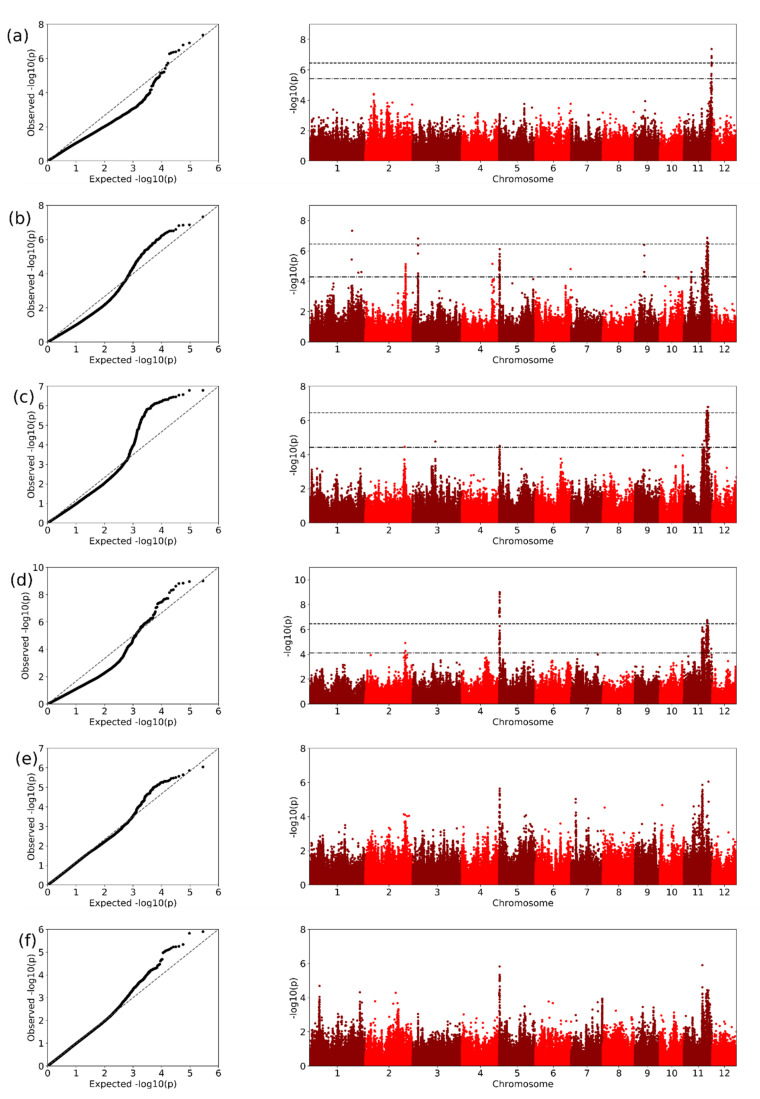

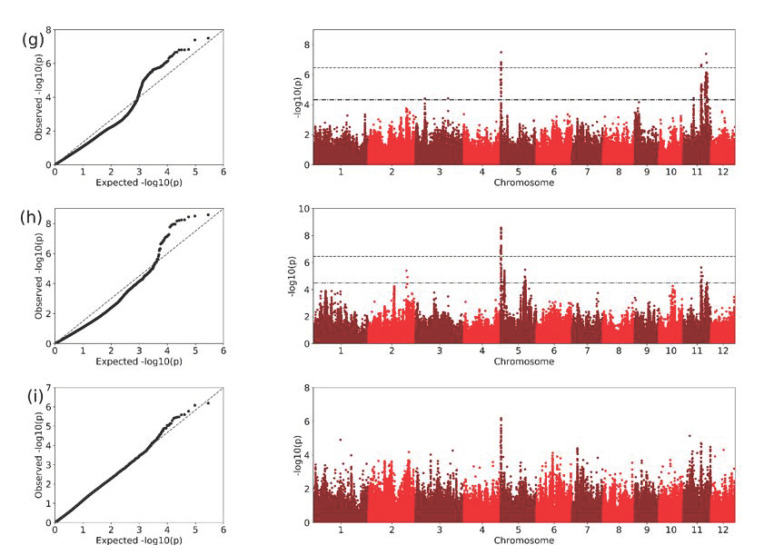
QQ and Manhattan plots from GWAS analyses highlighting significantly associated SNPs associating with bacterial leaf blight resistance for representative *Xoo* isolates. Figures (**a**–**i**), *Xoo* isolates: (**a**) 2XORE1-14; (**b**) 2XOST2-2; (**c**) 3XOBR2-6; (**d**) 4XORB4-5; (**e**) 59XOCRMS9-4; (**f**) 59XOCRMSO3-1-1; (**g**) 59XOCRPA20-10; (**h**) SP1-1; and (**i**) XONS3-2. Dot-dash and dashed lines represent FDR and bootstrap significance thresholds, respectively.

We used genome-wide association study (GWAS) methods in Tassel implementing an MLM model to identify loci associated with the resistance to *Xoo* isolates (Figure 5; Table 1 and Table 2). Using false discovery rate (FDR) evaluation, we identified 406 significantly associated SNPs across chromosomes 1–6, 8–10 and 11. Of these, 207 were contained within 147 MSU designated gene regions across chromosomes 1–6, 8, 9 and 11, identified across seven *Xoo* strains (meaning 13 strains yielded no associated SNPs) (Table S2a,b). Additionally, we also employed bootstrap evaluation which proved more conservative, identifying 51 significantly associated SNPs within 26 MSU annotated regions on chromosomes 3, 5 and 11.

Only one identified SNP, at LOC_Os05g01710, is situated within a known BLB resistance gene (*Xa5*) within the genome. However, 21 MSU designated genes are located within LD decay thresholds of previously cloned *R* genes on chromosomes 5 and 11. A further 127 MSU genes are found external to the identified LD blocks surrounding *R* genes on chromosomes 1–6, 8, 9 and 11. Furthermore, our analyses identified SNPs within MSU annotated genes on chromosomes 1, 2, 5 and 11 that have GOSlim classifications indicating they are functional regions involved in stress responses to external stimuli (Table S2). snpEff analyses indicated that four of these annotated genes feature alternative alleles that possess mutations predicted to confer “moderate” (i.e., functional) influence on their respective gene sequences (Table S3). Additionally, a further 199 significant loci were identified outside of the designated MSU gene loci on chromosomes 1–3, 5 and 8–11. Mostly these are on chromosomes 5 and 11 and generally constitute clusters of SNPs occupying regions where identified MSU genes are concentrated.

**Figure 6:**
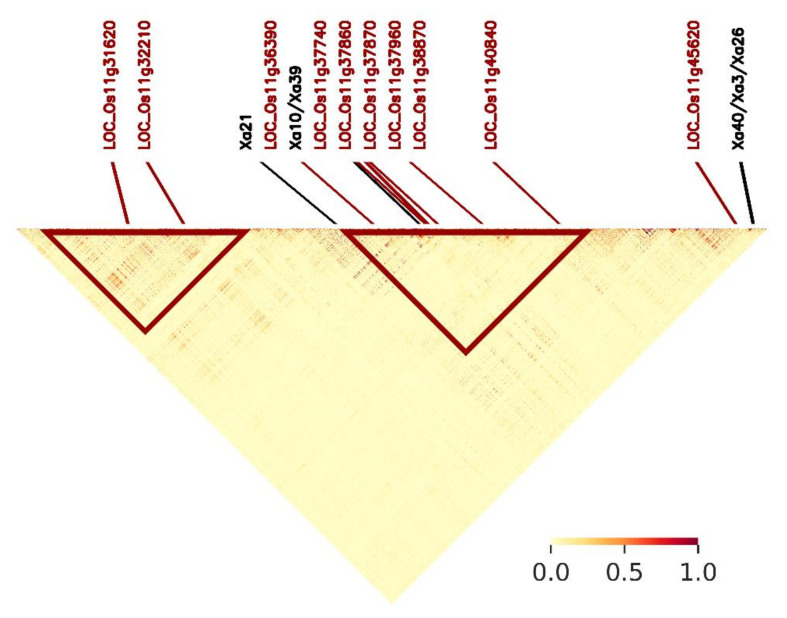
Linkage disequilibrium (LD) heatmap for chromosome 11 (region 17–29Mb) indicating chromosomal locations of six previously mapped R (*Xa*) genes and ten MSU annotated genes (LOC_Os prefix) that contain significantly associated SNPs highlighted by GWAS that are: (i) greater than LD decay threshold (61 Kb) away from *R* genes; and (ii) described by Go categorizations as having stress response functionality. A lack of red areas on the heatmap indicates the generally low levels of LD for this chromosome. Red triangles indicate densely clustered regions of significantly associated SNPs. Pointers show map positions (bottom) and relative position on chromosome within the region (top).

**Figure 7:**
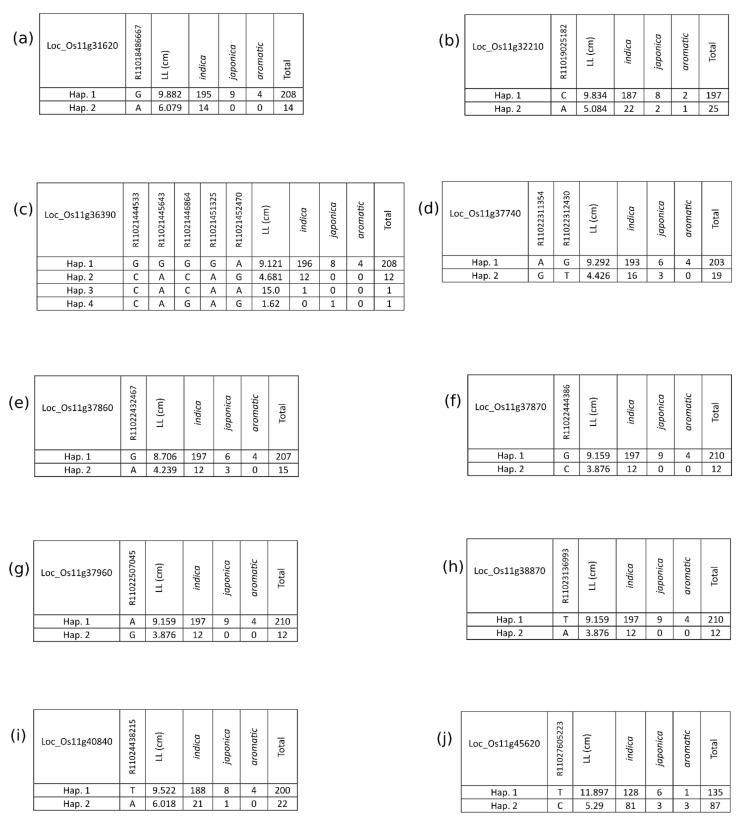
Tabular representation of alternative haplotypes determined by occurrence of significantly associated SNPs identified by GWAS analysis. (**a**–**j**) indicates specified MSU annotated genes.

**Figure 8:**
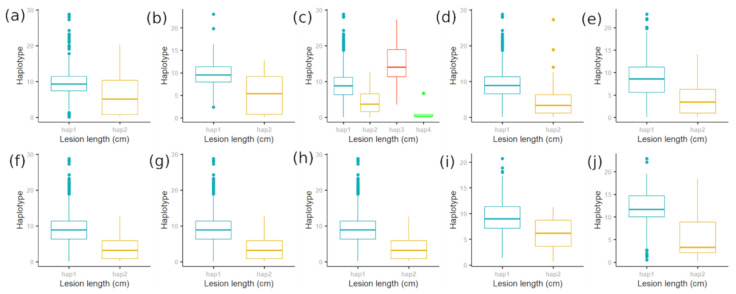
Boxplots indicating phenotypic responses in BLB resistance among rice accessions featuring alternative haplotypes determined by occurrence of significantly associated SNPs identified by GWAS analysis. (**a**) LOC_Os11g31620; (**b**) LOC_Os11g32210; (**c**) LOC_Os11g40840; (**d**) LOC_Os11g45620; (**e**) LOC_Os11g37860; (**f**) LOC_Os11g37870; (**g**) LOC_Os11g37960; (**h**) LOC_Os11g38870; (**i**) LOC_Os11g31620; and (**j**) LOC_Os11g31620.

Figure 6 shows the positions of 10 identified MSU genes on chromosome 11 within the 17–29 Mb region, including two genes (LOC_Os11g31620 and LOC_Os11g32210) that are entirely isolated from known *R* gene positions. Significantly associated SNPs largely appear within two clusters where the 10 MSU genes are predominantly situated. In general, these genes harbour haplotypes (Figure 7) that confer significantly different levels of BLB susceptibility (Figure 8; Table 3). We highlight the positions of further stress response genes (including other MSU genes containing significantly associated SNPs) in Figure S2.

Finally, significantly associated SNPs were found across seven *Xoo* isolates, meaning 13 *Xoo* isolates did not yield significantly associated loci. Individual MSU gene regions were found to contain significantly associated SNPs (FDR threshold) across a maximum of six *Xoo* isolates (Tables S2 and S3). Moreover, many MSU regions only contained significantly associated SNPs (FDR threshold) across a single *Xoo* isolate (e.g., on chromosomes 1 and 2). *Xoo* strains SP1-1, 2XOST2-2, 3XOBR2-6, 4XORB4-5, 59XOCRPA20-10 and 60XOCRMSY1 yielded between 4-79 MSU gene regions containing significant SNPs with 1–5 SNPs per gene region (Table 4).

## 3. Discussion

Since the 1970s, bacterial leaf blight has become one of the biggest rice production concerns in Asia. The problem seemingly exacerbated by modern agricultural techniques combined with anthropogenic environmental (e.g., climate) change. We conducted GWAS on a 222-accession panel of mostly Thai rice cultivars alongside some additional global varieties and comprising principally *indica* varieties, infected by representative Thai strains of 20 *Xoo* isolates to analyse the genetic basis of bacterial leaf blight (BLB) resistance in rice. We detected regions of significantly associated SNPs associated with *Xoo* resistance on chromosomes 1–6 and 8–11 (Table S2).

On chromosome 11, we identified hotspot regions that are substantial chromosomal distances away from previously reported *R* resistance genes suggesting the potential discovery of novel resistance genes against BLB. Notably, ten of these MSU designated genes highlighted on chromosome 11 have GOSlim classifications indicating that these regions are involved in stress responses from external environmental stimuli. In total, 241 of 406 significantly associated SNPs found across the genome are present on chromosome 11, mostly clustered on the 17–29 Mb region.

Rice accessions carrying the closely linked *Xa4* [60] and *Xa3/Xa26* genes on chromosome 11 exhibit broad resistance to numerous *Xoo* strains (NB these are also close to the more strain specific gene, *Xa43*). We did not find significant SNPs present within these gene regions although we identified significant SNPs within calculated linkage disequilibrium distance including within a nearby stress response gene (LOC_Os11g45620; Figure 6). It is also notable that our data do not corroborate findings from analyses of Philippines *Xoo* strains which reported many SNPs on chromosomes 6, 9 and 12 [24] (although the authors also reported associated SNPs on chromosome 11).

In addition to findings on chromosome 11, chromosome 5 features a cluster of significant SNPs close to the previously identified *R* gene, *xa5* [61], as well as a chromosomally distinct MSU designated gene associated with stress responses (LOC_Os05g05800; Figure S2b). Chromosome 5 features 92 of 406 significantly associated SNPs mostly clustered on the 0–6 Mb region. Chromosomes 1 and 2 also have clusters of significant SNPs including those from stress response genes. Previously, *R* genes have not been cloned on chromosomes 1 and 2. Chromosomes 1 and 2 feature 52 of the 406 significant SNPs across the chromosome meaning that chromosomes 1, 2, 5 and 11 account for *ca*. 94.8% of identified loci. Overall, it should also be noted that we further checked and failed to find identified significant SNPs located within 22 sweet gene regions known for associated susceptibility to *Xoo* (e.g., [62]).

In general, SNPs associated with BLB resistance appear contingent on the inoculated *Xoo* strain with only seven out of 20 strains eliciting significant loci from GWAS. Such race specificity has been noted for other reported R genes and QTLs [30,63,64]. This also holds true among the seven *Xoo* strains that elicited positive results, with efficacious loci often showing responses across only 2–3 strains (although SNPs on chromosome 11 are often associated with ≥5 *Xoo* strains, while SNPs on chromosomes 1 and 2 have single strain associations; Table S2). When considering our GWAS experiments were conducted against a large sample of Thai endemic *Xoo* strains (n = 20), this suggests that broad-scale effect genes may be generally elusive among global rice genotypes or only found at select loci (certainly among *indica* varieties).

These results concur with those of previous studies [24,54] in suggesting that broadscale, multi *Xoo* strain resistance may be difficult to identify in rice. One interesting point raised by Zhang et al. [54] is that BLB resistance may be particularly robust in *indica* varieties due to their tropical origins where outbreaks are most common due to favourable environmental factors for the survival, propagation, and infection of *Xoo* [2,63]. While our data primarily focusses on *indica* varieties, there is no obvious pattern suggesting that BLB resistance is more prevalent among our *indica* varieties when compared with *japonica* (Figure 7).

Our results, where significant SNPs were identified at best across six *Xoo* strains, combined with other studies suggest that the identification of ‘*silver bullet*’, broad-scale genomic resistance solutions to BLB pathogenicity is likely to prove evasive. Therefore, the most likely practical solutions should entail the development of multi-locus resistance rice varieties that provide greater antagonism to adaptive mutational responses across a range of *Xoo* strains that would be predicted to more readily undermine resistance in single-locus resistant varieties. Moreover, the tailoring of strategies that employ specific rice cultivars in explicit geographic regions with respect to localised presence of particular *Xoo* strains may be the optimal solution. From our data, the majority of significant loci that yield significant results against the most *Xoo* strains (typically ≥ 5; Table S2), are located in a cluster on chromosome 11 (from *ca*. 20.9 to 25 Mb) surrounding (either closely or at relatively large distances) around the resistance genes, *Xa10/Xa39* and *Xa21* (Figure 6). There is also a dense cluster of identified genes at around *ca*. 17.4 to 19.7 Mb that are associated with only a few *Xoo* strains. Notably, both these regions contain GOSlim classification stress response loci.

## 4. Materials and Methods

### 4.1. Plant Materials

A panel of rice germplasm consisting of 222 accessions previously subject to whole genome sequencing featuring a full reference database of variant SNP calls against the MSU rice reference genome (ver. 7; http://rice.plantbiology.msu.edu/pub/data/Eukaryotic_Projects/o_sativa/annotation_dbs/pseudomolecules/version_7.0/; accessed 10 April 2020).

### 4.2. BLB Screening

Our rice germplasm panel accessions were evaluated for BLB resistance against 20 different Thai *Xoo* isolates selected to comprehensively represent genetic and phenotypic variation of *Xoo* in Thailand (Table S1). Our rice germplasm panel accessions were evaluated for BLB resistance against 20 different Thai *Xoo* isolates selected to comprehensively represent genetic and phenotypic variation of *Xoo* in Thailand. The rice varieties IR24 and PYBB36 were used as susceptible and resistant standard checks, respectively. PYBB36 was developed by Rice Gene Discovery Unit (RGDU), Thailand, and is known to carry three BLB resistance genes, *xa5*, *Xa21*, and *xa33*. Bacterial inoculum were maintained on PSA (5 g peptone, 20 g sucrose, 3 g beef extract, and 15 g agar, adjusted to 1 litter with dH_2_O) medium for 72 h at 28 °C before application. The bacterial cells were suspended in sterile water at a density of 10^9^ cells/mL. Plants were grown under greenhouse condition at RGDU. The inoculation followed the clipping method outlined by Kauffman et al., [65]. For each accession (n = 222), two almost fully expanded leaves from four replicate plants for each of 20 *Xoo* isolates (n = 17,760 experimental plants) were removed and measured for lesion length (LL) at 14 days after inoculation or after the susceptibility check was complete. Plants were evaluated for BLB resistance by measuring the length of lesions (LL) based on the Standard Evaluation System (SES) recommended by IRRI (Table S1). Lesion lengths <5 cm were considered as resistant (R). >5–10 cm were considered as moderately resistant (MR), >10–15 cm were considered as moderately susceptible (MS), and >15 cm were considered as susceptible (S).

### 4.3. Estimation of Population Parameters

We conducted Principal Component Analysis (PCA) to (i) understand the genetic structure among the rice accession panel, and (ii) integrate with representative data from the IRRI 3000 Rice Genomes Project (http://iric.irri.org/resources/3000-genomes-project; accessed 11 March 2020) database, to establish what typed varieties our accession panel clusters against. Next, we conducted Linkage Disequilibrium (LD) assessment to evaluate the mean size of genomic linkage blocks within each chromosome. We thinned our VCF file in vcftools [66] so no SNPs were less than 1000 bp apart in order that the dataset was computationally manageable. Pairwise linkage between all remaining loci within chromosomes were conducted using the R library *LDheatmap* [67]. Outputted results were then passed to custom Python scripts for plotting by calculating mean LD within 100 bin divisions of total chromosome length.

### 4.4. Genome-Wide Association Study (GWAS) Methods

The association of SNP markers and bacterial leaf blight resistance was performed using TASSEL 5.0 [68]. A total number of 142,362 SNPs that met the filtering criteria (MAF >0.5 and missing data less than 30%) were used as genotype data while lesion lengths determined from leaves inoculated by each of 20 *Xoo* isolates were used as phenotypic data. A PCA was conducted to assess population structure and a kinship (K) matrix was created using centred identity-by-state to determine the familial relatedness between rice accessions. A mixed linear model [69] was used for performing GWAS by incorporating the K matrix along with the first three PCs using TASSEL 5.0.

From Tassel outputs we produced Python scripts to generate QQ-plots by plotting observed probabilities for each marker against the set of probabilities at which to evaluate the inverse distribution [70]. We also generated Manhattan plots to illustrate individual marker associations by plotting each marker against the negative logarithm of its GWAS-generated probability. For each plot we calculated the false discovery rate at 5% threshold [71] and the Bonferroni correction threshold also at 5% [72]. In order to better visualize and examine the identified SNPs and genomic regions from our GWAS analyses we used customized Python programming to integrate our GWAS results against the MSU pseudomolecule genomic regions database hosted by the Rice Genome Annotation Project [73]. Our program (https://github.com/ctdarwell/TASSELmanip) facilitates clear presentation of all identified significantly associated SNPs (using FDR and Bonferroni) within a GWAS analysis, permitting visualisation of which significant MSU genomic regions are identified for each inspected *Xoo* strain allowing easy assessment of potential loci that may confer broad multi-strain resistance potential. Main results are displayed in tabular form while other output files are generated, including a table that indicates MSU annotated function of all genomic regions containing significantly associated SNPs.

### 4.5. Additional Genomic Evaluation

To evaluate whether the significantly associated SNPs that we identified are likely to have independent functional contribution to resistance phenotypes against bacterial leaf blight, we conducted several further analyses. First, GOSlim (http://rice.plantbiology.msu.edu/downloads_gad.shtml; accessed 2 July 2020) annotations for genes containing significant loci identified by GWAS were searched to identify loci that have potential functional influence on bacterial leaf blight resistance. In particular, we focused on loci that are further away than the average LD influence block size (according to an r^2^ criterion of 0.2) for a given chromosome, in order to highlight regions that may potentially harbour novel genes underpinning BLB resistance. Further, we only selected loci whose GOSlim annotations are categorized as stress response genes that therefore most likely predict novel candidate resistance genes. Finally, to further determine whether allelic variation found in these loci is likely to have functional influence on bacterial leaf blight resistance, we use snpEff  [74] to predict whether significantly associated SNPs have a robust predicted effect on functional outcomes. Our findings indicated that the most likely source of novel BLB resistance genes is chromosome 11 due to its yielding of the greatest number of significantly associated SNPs most of which are on unlinked regions of the chromosome from *R* genes. For this chromosome, we conducted further analyses on identified stress response gene sequence data to identify haplotypes based on presence/absence of significantly associated alleles. We describe this haplotypic variation and conducted anova analysis on its correlation with phenotypic variation in BLB resistance across accessions.

## 5. Conclusions

Our study provides new insight into the genomic basis of bacterial blight resistance in rice. We anticipate that our findings will be useful for the development of BLB resistant rice lines in research programs in both Thailand and also other locations. Our focal rice accessions that we have mapped and indicated novel loci will hopefully provide more available material in breeding programs aimed at developing BLB resistance in different rice growing regions. Future research should focus on validating the effects of our identified candidate markers and characterizing their functional effects with respect to BLB resistance.

## Figures and Tables

**Table 1:** SNPs associated with BLB resistance across seven *Xoo* isolates.

*Xoo* Strain	Chr	Position (bp)	−log10 (*p*-Value)	SNP Genotypes	MAF (%)	Marker r^2^	Genetic Var	Residual Var	Heritability
SP1-1	2	28895260	5.4	G,T	11.11	0.12	3.77	7.15	0.35
	5	372359	8.58	A,C	8.78	0.19	3.77	7.15	0.35
	11	17828845	5.63	C,T	7.22	0.14	3.77	7.15	0.35
2XOST2-2	1	35320544	7.33	C,A	5.91	0.16	4.5	5.44	0.45
	2	30161196	5.13	T,G	13.44	0.13	4.5	5.44	0.45
	3	3379824	6.81	A,G	5.52	0.16	4.5	5.44	0.45
	4	30623393	5.15	T,C	6.15	0.11	4.5	5.44	0.45
	5	457765	6.12	T,G	4.59	0.13	4.5	5.44	0.45
	6	30380545	4.8	C,A	6.19	0.1	4.5	5.44	0.45
	9	10748870	6.4	T,G	7.04	0.14	4.5	5.44	0.45
	11	22311354	6.86	A,G	8.72	0.14	4.5	5.44	0.45
3XOBR2-6	2	29390164	4.46	C,T	23.53	0.1	2.13	10.46	0.17
	3	16299521	4.77	G,C	5.97	0.1	2.13	10.46	0.17
	5	463077	4.51	A,G	7.43	0.09	2.13	10.46	0.17
	11	23204208	6.8	C,G	6.82	0.17	2.13	10.46	0.17
2XORE1-14	11	27582018	7.37	A,G	30.48	0.17	7.03	12.16	0.37
4XORB4-5	2	29791617	4.91	C,A	16.48	0.11	3.55	7.82	0.31
	5	461986	9.01	G,A	4.5	0.2	3.55	7.82	0.31
	11	22258758	6.75	G,A	5.82	0.16	3.55	7.82	0.31
59XOCRPA20-10	3	26795267	4.43	T,C	43.85	0.1	5.31	10.88	0.33
	5	461986	7.5	G,A	4.5	0.17	5.31	10.88	0.33
	11	22311354	7.39	A,G	8.72	0.17	5.31	10.88	0.33
60XOCRMSY1	1	33302431	5.17	T,C	6.53	0.1	10.28	9.19	0.53
	3	16327075	4.54	T,G	7.27	0.11	10.28	9.19	0.53
	5	461986	7.31	G,A	4.5	0.16	10.28	9.19	0.53
	8	4006324	4.53	G,A	11.56	0.09	10.28	9.19	0.53
	10	15740836	4.7	A,G	5.83	0.1	10.28	9.19	0.53
	11	23617456	6.85	A,T	9.9	0.15	10.28	9.19	0.53

**Table 2:** Linkage clusters within 5Mb blocks across seven *Xoo* isolates. Clusters are delineated according to LD decay values for each chromosome.

Chromosome	Representative SNP Position (bp)	*Xoo* Strain	No. of Linkage Blocks	MSU ID: Annotation
1	35320544	2XOST2-2; 60XOCRMSY1	5	LOC_Os01g57082: insulin-degrading enzyme, putative, expressed; LOC_Os01g57590: expressed protein; LOC_Os01g59450: ZOS1-13-C2H2 zinc finger protein, expressed; LOC_Os01g60440: HEAT repeat family protein, putative, expressed; LOC_Os01g60660: methionyl-tRNA synthetase, putative, expressed; LOC_Os01g61110: ulp1 protease family, C-terminal catalytic domain containing protein, expressed; LOC_Os01g61120: expressed protein; LOC_Os01g61335: expressed protein; LOC_Os01g61440: expressed protein; LOC_Os01g61590: CAMK_CAMK_like.1—CAMK includes calcium/calmodulin dependent protein kinases, expressed;
1	35320544	2XOST2-2; 60XOCRMSY1	4	LOC_Os01g66490: no apical meristem protein, putative, expressed; LOC_Os01g66860: serine/threonine protein kinase, putative, expressed; LOC_Os01g70340: expressed protein; LOC_Os01g71960: endonuclease, putative, expressed;
2	28895260	SP1-1; 2XOST2-2; 3XOBR2-6; 4XORB4-5	3	LOC_Os02g47310: Cyclopropane-fatty-acyl-phospholipid synthase, putative, expressed; LOC_Os02g48690: expressed protein; LOC_Os02g48730: rho GDP-dissociation inhibitor 1, putative, expressed; LOC_Os02g48830: microtubule associated protein, putative, expressed; LOC_Os02g48990: phosphatidylinositol transfer, putative, expressed; LOC_Os02g49140: glycosyltransferase, putative, expressed; LOC_Os02g49180: RNA polymerase subunit, putative, expressed; LOC_Os02g49230: CCT/B-box zinc finger protein, putative, expressed; LOC_Os02g49360: RNA methyltransferase domain-containing protein 2, putative, expressed;
3	3379824	2XOST2-2; 59XOCRPA20-10	2	LOC_Os03g06680: plant-specific domain TIGR01615 family protein, expressed; LOC_Os03g06710: PPR repeat domain containing protein, putative, expressed; LOC_Os03g12760: helix-loop-helix DNA-binding domain containing protein, expressed;
3	3379824	3XOBR2-6; 60XOCRMSY1	1	
3	3379824	59XOCRPA20-10	1	LOC_Os03g47380: expressed protein;
4	30623393	2XOST2-2	1	LOC_Os04g51690: glycosyl hydrolase family 47 domain contain protein, expressed;
5	461986	SP1-1; 2XOST2-2; 3XOBR2-6; 4XORB4-5; 59XOCRPA20-10; 60XOCRMSY1	3	LOC_Os05g01030: phospholipid-transporting ATPase, putative, expressed; LOC_Os05g01060: expressed protein; LOC_Os05g01490: ras-related protein, putative, expressed; LOC_Os05g01500: tubulin-specific chaperone E, putative, expressed; LOC_Os05g01590: heat shock protein DnaJ, putative, expressed; LOC_Os05g01610: FYVE zinc finger domain containing protein, expressed; LOC_Os05g01620: OsFBX155—F-box domain containing protein, expressed; LOC_Os05g01690: expressed protein; LOC_Os05g01700: ABC transporter, ATP-binding protein, putative, expressed; LOC_Os05g01710: transcription initiation factor IIA gamma chain, putative, expressed; LOC_Os05g01730: drought induced 19 protein, putative, expressed; LOC_Os05g01750: TruB family pseudouridylate synthase, putative, expressed; LOC_Os05g01760: lysine ketoglutarate reductase trans-splicing related 1, putative, expressed; LOC_Os05g01780: STE_PAK_Ste20++TranslationKinase_Slob_Wnk.1 - STE kinases include homologs to sterile 7, sterile 11 and sterile 20 from yeast, expressed; LOC_Os05g01790: expressed protein; LOC_Os05g01810: xylem cysteine proteinase 2 precursor, putative, expressed; LOC_Os05g01910: pumilio-family RNA binding protein, putative, expressed; LOC_Os05g05700: cullin, putative, expressed; LOC_Os05g05770: hypothetical protein; LOC_Os05g05790: double-stranded RNA binding motif containing protein, expressed; LOC_Os05g05800: OsFBL21—F-box domain and LRR containing protein, expressed; LOC_Os05g05840: tRNA synthetase class II core domain containing protein, expressed; LOC_Os05g05880: retrotransposon protein, putative, unclassified, expressed; LOC_Os05g05910: retrotransposon protein, putative, unclassified, expressed; LOC_Os05g05950: TOC159, putative, expressed; LOC_Os05g06014: expressed protein; Xa5_Os05g01120: cytochrome P450, putative, expressed;
5	461986	SP1-1	3	LOC_Os05g37500: expressed protein; LOC_Os05g37830: expressed protein; LOC_Os05g38270: regulator of chromosome condensation, putative, expressed;
5	461986	60XOCRMSY1	1	
6	30380545	2XOST2-2	1	LOC_Os06g50170: BRE, putative, expressed;
8	4006324	60XOCRMSY1	1	LOC_Os08g07170: cytokinin-O-glucosyltransferase 2, putative, expressed; LOC_Os08g07380: retrotransposon protein, putative, unclassified, expressed;
9	10748870	2XOST2-2	1	LOC_Os09g17650: expressed protein;
10	15740836	60XOCRMSY1	1	
11	22311354	2XOST2-2; 59XOCRPA20-10	3	LOC_Os11g13750: expressed protein;
11	22311354	SP1-1; 2XOST2-2; 3XOBR2-6; 4XORB4-5; 59XOCRPA20-10; 60XOCRMSY1	17	LOC_Os11g30370: OsSPL19 - SBP-box gene family member, expressed; LOC_Os11g30560: dehydrogenase/reductase, putative, expressed; LOC_Os11g30600: hypothetical protein; LOC_Os11g30620: expressed protein; LOC_Os11g30740: transposon protein, putative, CACTA, En/Spm sub-class, expressed; LOC_Os11g30770: expressed protein; LOC_Os11g30790: expressed protein; LOC_Os11g30860: retrotransposon protein, putative, Ty3-gypsy subclass, expressed; LOC_Os11g30930: expressed protein; LOC_Os11g30940: retrotransposon protein, putative, unclassified, expressed; LOC_Os11g30960: retrotransposon protein, putative, unclassified, expressed; LOC_Os11g31050: retrotransposon protein, putative, unclassified, expressed; LOC_Os11g31090: transferase family protein, putative, expressed; LOC_Os11g31500: ATP binding protein, putative, expressed; LOC_Os11g31620: OsFBL55—F-box domain and LRR containing protein, expressed; LOC_Os11g31650: expressed protein; LOC_Os11g31670: retrotransposon protein, putative, unclassified, expressed; LOC_Os11g31690: expressed protein; LOC_Os11g31950: expressed protein; LOC_Os11g32210: jacalin-like lectin domain containing protein, expressed; LOC_Os11g32320: CCB1, putative, expressed; LOC_Os11g32340: hypothetical protein; LOC_Os11g32360: expressed protein; LOC_Os11g32369: expressed protein; LOC_Os11g32390: expressed protein; LOC_Os11g32410: expressed protein; LOC_Os11g32530: retrotransposon protein, putative, unclassified, expressed; LOC_Os11g32570: expressed protein; LOC_Os11g33190: OsFBX422 - F-box domain containing protein, expressed; LOC_Os11g35870: RWD domain containing protein, expressed; LOC_Os11g36050: prefoldin subunit, putative, expressed; LOC_Os11g36060: THUMP domain-containing protein, putative, expressed; LOC_Os11g36070: expressed protein; LOC_Os11g36090: receptor kinase, putative, expressed; LOC_Os11g36140: receptor-like protein kinase 2 precursor, putative, expressed; LOC_Os11g36180: receptor kinase, putative, expressed; LOC_Os11g36340: lymphoid organ expressed yellow head virus receptor protein, putative, expressed; LOC_Os11g36350: OsFBDUF50—F-box and DUF domain containing protein, expressed; LOC_Os11g36390: RFC1 - Putative clamp loader of PCNA, replication factor C subunit 1, expressed; LOC_Os11g37000: heat shock protein DnaJ, putative, expressed; LOC_Os11g37090: pumilio-family RNA binding repeat domain containing protein, expressed; LOC_Os11g37100: expressed protein; LOC_Os11g37130: mttA/Hcf106 family protein, putative, expressed; LOC_Os11g37140: expressed protein; LOC_Os11g37260: SEY1, putative, expressed; LOC_Os11g37300: OsFBDUF53—F-box and DUF domain containing protein, expressed; LOC_Os11g37330: pentatricopeptide repeat domain containing protein, putative, expressed; LOC_Os11g37510: ribosomal protein L4, putative, expressed; LOC_Os11g37670: expressed protein; LOC_Os11g37680: expressed protein; LOC_Os11g37690: TBC domain containing protein, expressed; LOC_Os11g37700: pleiotropic drug resistance protein, putative, expressed; LOC_Os11g37730: glutathione S-transferase, N-terminal domain containing protein, expressed; LOC_Os11g37740: stripe rust resistance protein Yr10, putative, expressed;
11	22311354	SP1-1; 2XOST2-2; 3XOBR2-6; 2XORE1-14; 4XORB4-5; 59XOCRPA20-10; 60XOCRMSY1	12	LOC_Os11g37860: stripe rust resistance protein Yr10, putative, expressed; LOC_Os11g37870: stripe rust resistance protein Yr10, putative, expressed; LOC_Os11g37890: NAD dependent epimerase/dehydratase family protein, putative, expressed; LOC_Os11g37960: WIP4—Wound-induced protein precursor, expressed; LOC_Os11g38010: targeting protein for Xklp2, putative, expressed; LOC_Os11g38020: GTPase of unknown function domain containing protein, putative, expressed; LOC_Os11g38040: expressed protein; LOC_Os11g38050: phosphoesterase family protein, putative, expressed; LOC_Os11g38140: OsFBDUF58—F-box and DUF domain containing protein, expressed; LOC_Os11g38270: hypothetical protein; LOC_Os11g38620: expressed protein; LOC_Os11g38630: expressed protein; LOC_Os11g38640: expressed protein; LOC_Os11g38670: DEAD-box ATP-dependent RNA helicase, putative, expressed; LOC_Os11g38800: zinc finger family protein, putative, expressed; LOC_Os11g38810: mannose-6-phosphate isomerase, putative, expressed; LOC_Os11g38870: helix-loop-helix DNA-binding domain containing protein, expressed; LOC_Os11g38900: histone-lysine N-methyltransferase, H3 lysine-9 specific SUVH1, putative, expressed; LOC_Os11g38970: expressed protein; LOC_Os11g39360: pentatricopeptide repeat domain containing protein, putative, expressed; LOC_Os11g39540: 14-3-3 protein, putative, expressed; LOC_Os11g39650: WD domain, G-beta repeat domain containing protein, expressed; LOC_Os11g40200: expressed protein; LOC_Os11g40590: DUF1399 containing protein, putative, expressed; LOC_Os11g40840: receptor-like protein kinase 2 precursor, putative, expressed; LOC_Os11g44910: DEAD-box ATP-dependent RNA helicase, putative, expressed;
11	22311354	2XORE1-14	3	LOC_Os11g45290: retrotransposon protein, putative, unclassified, expressed; LOC_Os11g45580: embryogenesis transmembrane protein, putative, expressed; LOC_Os11g45590: transposon protein, putative, CACTA, En/Spm sub-class, expressed; LOC_Os11g45620: rust-resistance protein Lr21, putative, expressed.

**Table 3:** Anova results of BLB resistance assays among different haplotypes determined by presence/absence of significantly associated SNPs contained within MSU gene boundaries. Bold indicates significance while *** indicates significance at the <0.0001 level.

MSU Locus	Haplotype Comparison	Mean Diffs	Lower CI (95%)	Upper CI (95%)	Adjusted P
LOC_Os11g31620	Hap1-Hap2	−3.803	−5.44	−2.167353	6.40E-06 ***
LOC_Os11g32210	Hap1-Hap2	−4.750	−6.037	−3.462886	0 ***
LOC_Os11g36390	Hap1-Hap2	−4.44	−5.858	−3.021265	0 ***
	Hap1-Hap3	5.879	1.129	10.62905	0.008 **
	Hap1-Hap4	−7.501	−12.251	−2.75095	3E-04 ***
	Hap2-Hap3	10.319	5.383	15.253991	5E-07 ***
	Hap2-Hap4	−3.061	−7.997	1.873991	0.3812906
	Hap3-Hap4	−13.38	−20.081	−6.678551	2E-06 ***
LOC_Os11g37740	Hap1-Hap2	−4.866	−5.726	−4.00688	0 ***
LOC_Os11g37860	Hap1-Hap2	−4.467	−5.671	−3.263031	0 ***
LOC_Os11g37870	Hap1-Hap2	−5.283	−6.363	−4.202503	0 ***
LOC_Os11g37960	Hap1-Hap2	−5.283	−6.363	−4.202503	0 ***
LOC_Os11g38870	Hap1-Hap2	−5.283	−6.363	−4.202503	0 ***

**Table 4:** Summary table of MSU designated gene regions containing significantly associated SNPs according to *Xoo* strain inoculation.

*Xoo* Strain	No. of MSU Genes Affected	Min. No. SNPs Per Gene	Max. No. SNPs Per Gene
SP1-1	33	1	4
2XOST2-2	62	1	4
3XOBR2-6	44	1	5
2XORE1-14	4	1	3
4XORB4-5	79	1	4
59XOCRPA20-10	54	1	3
60XOCRMSY1	66	1	3

## Data Availability

The data supporting the conclusions of this article are included within the article and its additional files. Python programming files available at: https://github.com/ctdarwell/TASSELmanip.

## Supplementary Materials

The following are available online at https://www.mdpi.com/2223-7747/10/3/518/s1. Figure S1. See “Supplementary File 1.docx”. Figure S2. See “Supplementary File 1.docx”. Table S1. See “Supplementary File 1.docx”. Table S2a; see “Supplementary File 1.docx” for title, and “Supplementary File 2.pdf” for Python program generated output. Table S2b; see “Supplementary File 1.docx” for title and “Supplementary File 3.pdf” for Python program generated output. Table S3. See “Supplementary File 1.docx”. Table S4. See “Supplementary File 1.docx”. Figure 2. See “Supplementary data file 1.csv” for phenotypic data.
