# Supplementary material for: Identification of Bacterial Blight Resistance Loci in Rice (Oryza sativa L.) against Diverse Xoo Thai Strains by Genome-Wide Association Study
Source: Plants (Basel). 2021 Mar 10;10(3):518. doi: 10.3390/plants10030518 (PMC8001028; doi:10.3390/plants10030518)
Supplement: Supplementary file 1 [file plants-10-00518-s001.pdf]

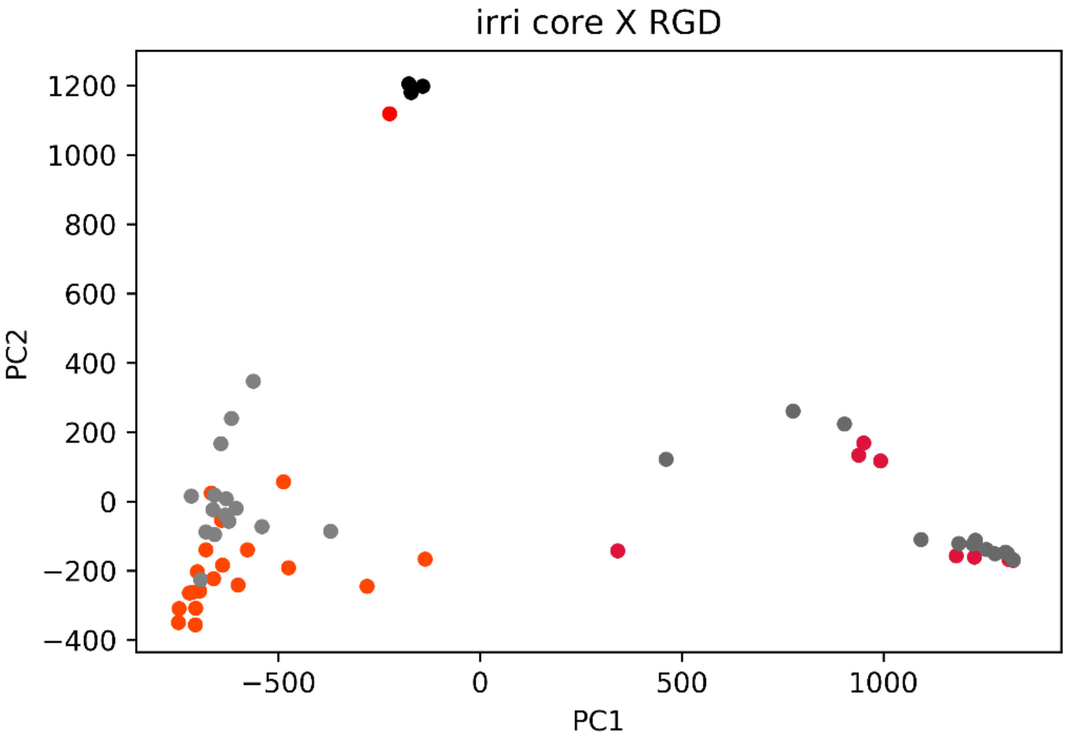

Figure S1. PCA analysis of representative Thai rice accessions alongside representative samples of global varieties downloaded from the International Rice research Institute (IRRI; irri.org).

(**a**)

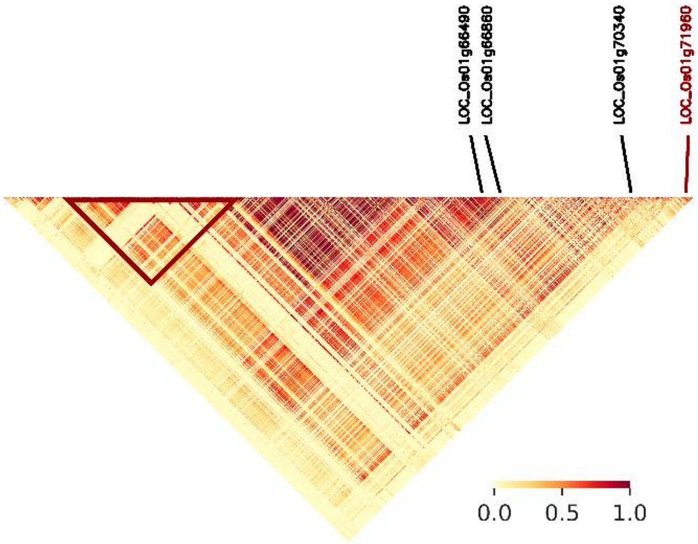

(**b**)

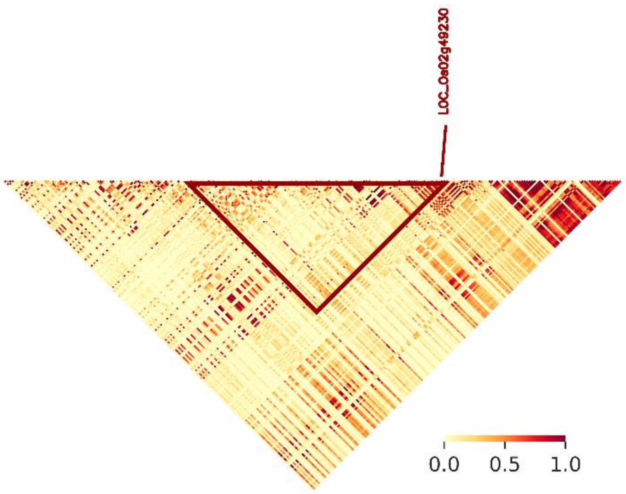

(**c**)

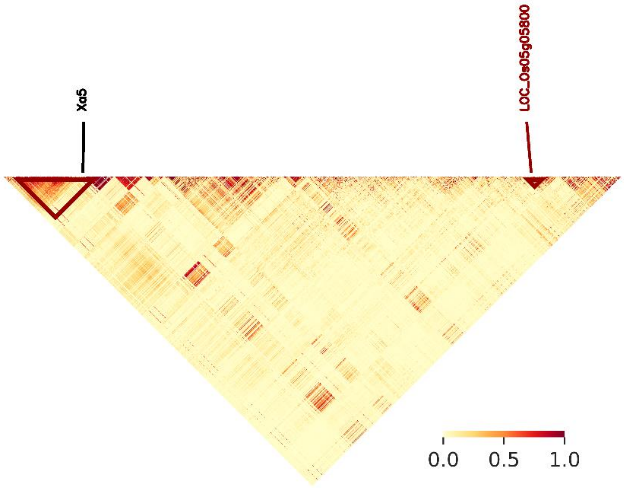

Figure S2. LD heatmaps of chromosomal regions harbouring large numbers of significantly segregating SNPs and indicating positions of notable MSU genes. (**a**) chromosome 1 – MSU LOC_Os01g71960 (red) is a known stress response gene, other identified genes (indicated individually in black or as a cluster with a red triangle) containing significantly segregating SNPs are shown; (**b**) chromosome 2 – LOC_Os02g49230 is a stress response gene clustered among other genes containing significantly segregating SNPs; and (**c**) chromosome 5 – LOC_Os05g58000 is a stress response gene, while *Xa* gene, *Xa5*, is clustered among other genes containing significantly segregating SNPs (red triangle).

Table S1. Resistance versus susceptibility assignments for 20 *Xoo* isolates. The column "Reaction" indicates assays of a set of 11 rice varieties that are introgression lines containing individual *Xa* genes. Pathogenicity of each line is assayed against each *Xoo* isolate (R = resistant, S = susceptible). Individual *Xa* genes contained within the 11 lines are: *Xa1*, *Xa3*, *Xa4*, *xa5*, *Xa7*, *xa8*, *Xa10*, *Xa11*, *xa13*, *Xa14* and *Xa21*.

Group No.	Isolate	Reaction	Year	Location
1	SK2-3	SSSSSSSSRSS	2551	Sukhothai
2	2XOST2-2	SRRRRSSSSSS	2555	Sukhothai
3	60XOCRMS Y-1	SSSRRRSRSSS	2560	Chiang Rai
4	XONS2-1	SSRRSSSSSSS	2555	Nakhon Si Thammarat
5	SP1-1	SSSRSSSSSSS	2551	Suphan Buri
6	3XOBR5-1	SSRRRSSRSSR	2555	Buri Ram
7	2XORE1-14	SSRRRSRRSSR	2555	Roi Et

8	XONS3-2	SSSRSSSSSRS	2555	Nakhon Si Thammarat
9	3XOBR2-6	SSSRSSSSSSR	2555	Buri Ram
10	59XOCRMS9-4	SSSSSSSRRSS	2559	Chiang Rai
11	59XOCRMSO3-1-1	SSSSSSSRRSR	2559	Chiang Rai
12	59XOCRMS1-9	SSSRSSSSSSS	2559	Chiang Rai
13	4XORB4-5	SSSRRSSSSSS	2555	Ratchaburi
14	59 XOCRPA20-10	SSSSSSSRRSS	2559	Chiang Rai
15	60XOCRPA19-9	SSSSSRRSRSS	2560	Chiang Rai
16	60XOCRPA3-6	SSSSRRSRRSR	2560	Chiang Rai
17	60XOCRPA15-5	SSSSSSSRSSS	2560	Chiang Rai
18	60XOCRPA25-9	SSSSSSSSRSR	2560	Chiang Rai
19	59XOCRPA22-1	SSSSSSSSRSS	2559	Chiang Rai
20	60XOCRPA27-8	SSSSSSSSSSS	2560	Chiang Rai

Table S2a. Table indicating identified MSU gene regions that contain significantly associated SNPs using False Discovery Rate (FDR) thresholds. Column headers indicate *Xoo* isolate where significance was reached. Different colours represent chromosomal location. See “Supplementary File 2.pdf”.

Table S2b. Table indicating identified MSU gene regions that contain significantly associated SNPs using bootstrap thresholds. Column headers indicate *Xoo* isolate where significance was reached. Different colours represent chromosomal location. See “Supplementary File 3.pdf”.

Table S3. GOSLIM annotations for identified MSU gene regions containing significantly segregating SNPs.

Model	Putative Function	GOSlim ID	GO Name
LOC_Os11g40840.1	receptor-like protein kinase 2 precursor, putative, expressed	GO:0009653	anatomical structure morphogenesis
LOC_Os11g30790.1	expressed protein	GO:0005488	binding
LOC_Os11g31620.1	OsFBL55 - F-box domain and LRR containing protein, expressed	GO:0005488	binding
LOC_Os11g35870.1	RWD domain containing protein, expressed	GO:0005488	binding
LOC_Os11g35870.1	RWD domain containing protein, expressed	GO:0005488	binding
LOC_Os11g35870.1	RWD domain containing protein, expressed	GO:0005488	binding
LOC_Os11g35870.1	RWD domain containing protein, expressed	GO:0005488	binding
LOC_Os11g37090.1	pumilio-family RNA binding repeat domain containing protein, expressed	GO:0005488	binding
LOC_Os11g37100.1	expressed protein	GO:0005488	binding
LOC_Os11g38620.1	expressed protein	GO:0005488	binding
LOC_Os11g38620.1	expressed protein	GO:0005488	binding
LOC_Os11g38630.1	expressed protein	GO:0005488	binding
LOC_Os11g38640.1	expressed protein	GO:0005488	binding
LOC_Os11g38800.1	zinc finger family protein, putative, expressed	GO:0005488	binding
LOC_Os11g38800.1	zinc finger family protein, putative, expressed	GO:0005488	binding

LOC_Os11g13750.1	expressed protein	GO:0008150	biological_process
LOC_Os11g30790.1	expressed protein	GO:0008150	biological_process
LOC_Os11g31500.1	ATP binding protein, putative, expressed	GO:0008150	biological_process
LOC_Os11g31950.1	expressed protein	GO:0008150	biological_process
LOC_Os11g32360.1	expressed protein	GO:0008150	biological_process
LOC_Os11g32360.1	expressed protein	GO:0008150	biological_process
LOC_Os11g36060.1	THUMP domain-containing protein, putative, expressed	GO:0008150	biological_process
LOC_Os11g36060.1	THUMP domain-containing protein, putative, expressed	GO:0008150	biological_process
LOC_Os11g36070.3	expressed protein	GO:0008150	biological_process
LOC_Os11g36070.3	expressed protein	GO:0008150	biological_process
LOC_Os11g36070.3	expressed protein	GO:0008150	biological_process
LOC_Os11g36070.3	expressed protein	GO:0008150	biological_process
LOC_Os11g36070.3	expressed protein	GO:0008150	biological_process
LOC_Os11g36070.3	expressed protein	GO:0008150	biological_process
LOC_Os11g36070.3	expressed protein	GO:0008150	biological_process
LOC_Os11g36350.1	OsFBDUF50 - F-box and DUF domain containing protein, expressed	GO:0008150	biological_process
LOC_Os11g37300.1	OsFBDUF53 - F-box and DUF domain containing protein, expressed	GO:0008150	biological_process
LOC_Os11g38010.1	targeting protein for Xklp2, putative, expressed	GO:0008150	biological_process
LOC_Os11g38140.1	OsFBDUF58 - F-box and DUF domain containing protein, expressed	GO:0008150	biological_process
LOC_Os11g38870.1	helix-loop-helix DNA-binding domain containing protein, expressed	GO:0008150	biological_process
LOC_Os11g38870.1	helix-loop-helix DNA-binding domain containing protein, expressed	GO:0008150	biological_process
LOC_Os11g40200.1	expressed protein	GO:0008150	biological_process
LOC_Os11g40590.3	DUF1399 containing protein, putative, expressed	GO:0008150	biological_process
LOC_Os11g40590.3	DUF1399 containing protein, putative, expressed	GO:0008150	biological_process
LOC_Os11g40590.3	DUF1399 containing protein, putative, expressed	GO:0008150	biological_process
LOC_Os11g30370.1	OsSPL19 - SBP-box gene family member, expressed	GO:0009058	biosynthetic process

LOC_Os11g31090.1	transferase family protein, putative, expressed	GO:0009058	biosynthetic process
LOC_Os11g36390.1	RFC1 - Putative clamp loader of PCNA, replication factor C subunit 1, expressed	GO:0009058	biosynthetic process
LOC_Os11g37330.2	pentatricopeptide repeat domain containing protein, putative, expressed	GO:0009058	biosynthetic process
LOC_Os11g37330.2	pentatricopeptide repeat domain containing protein, putative, expressed	GO:0009058	biosynthetic process
LOC_Os11g37890.1	NAD dependent epimerase/dehydratase family protein, putative, expressed	GO:0009058	biosynthetic process
LOC_Os11g37890.1	NAD dependent epimerase/dehydratase family protein, putative, expressed	GO:0009058	biosynthetic process
LOC_Os11g37890.1	NAD dependent epimerase/dehydratase family protein, putative, expressed	GO:0009058	biosynthetic process
LOC_Os11g38870.1	helix-loop-helix DNA-binding domain containing protein, expressed	GO:0009058	biosynthetic process
LOC_Os11g38870.1	helix-loop-helix DNA-binding domain containing protein, expressed	GO:0009058	biosynthetic process
LOC_Os11g37960.1	WIP4 - Wound-induced protein precursor, expressed	GO:0030246	carbohydrate binding
LOC_Os11g31090.1	transferase family protein, putative, expressed	GO:0005975	carbohydrate metabolic process
LOC_Os11g37330.2	pentatricopeptide repeat domain containing protein, putative, expressed	GO:0005975	carbohydrate metabolic process
LOC_Os11g37330.2	pentatricopeptide repeat domain containing protein, putative, expressed	GO:0005975	carbohydrate metabolic process
LOC_Os11g38810.1	mannose-6-phosphate isomerase, putative, expressed	GO:0005975	carbohydrate metabolic process
LOC_Os11g38810.1	mannose-6-phosphate isomerase, putative, expressed	GO:0005975	carbohydrate metabolic process
LOC_Os11g36390.1	RFC1 - Putative clamp loader of PCNA, replication factor C subunit 1, expressed	GO:0009056	catabolic process
LOC_Os11g37690.1	TBC domain containing protein, expressed	GO:0009056	catabolic process
LOC_Os11g37700.1	pleiotropic drug resistance protein, putative, expressed	GO:0009056	catabolic process
LOC_Os11g38050.1	phosphoesterase family protein, putative, expressed	GO:0009056	catabolic process
LOC_Os11g38620.1	expressed protein	GO:0009056	catabolic process
LOC_Os11g38620.1	expressed protein	GO:0009056	catabolic process
LOC_Os11g38630.1	expressed protein	GO:0009056	catabolic process
LOC_Os11g38640.1	expressed protein	GO:0009056	catabolic process

LOC_Os11g30560.1	dehydrogenase/reductase, putative, expressed	GO:0003824	catalytic activity
LOC_Os11g31620.1	OsFBL55 - F-box domain and LRR containing protein, expressed	GO:0003824	catalytic activity
LOC_Os11g37890.1	NAD dependent epimerase/dehydratase family protein, putative, expressed	GO:0003824	catalytic activity
LOC_Os11g37890.1	NAD dependent epimerase/dehydratase family protein, putative, expressed	GO:0003824	catalytic activity
LOC_Os11g37890.1	NAD dependent epimerase/dehydratase family protein, putative, expressed	GO:0003824	catalytic activity
LOC_Os11g38810.1	mannose-6-phosphate isomerase, putative, expressed	GO:0003824	catalytic activity
LOC_Os11g38810.1	mannose-6-phosphate isomerase, putative, expressed	GO:0003824	catalytic activity
LOC_Os11g30560.1	dehydrogenase/reductase, putative, expressed	GO:0005623	cell
LOC_Os11g32210.1	jacalin-like lectin domain containing protein, expressed	GO:0005623	cell
LOC_Os11g37860.1	stripe rust resistance protein Yr10, putative, expressed	GO:0005623	cell
LOC_Os11g37960.1	WIP4 - Wound-induced protein precursor, expressed	GO:0005623	cell
LOC_Os11g38870.1	helix-loop-helix DNA-binding domain containing protein, expressed	GO:0007154	cell communication
LOC_Os11g38870.1	helix-loop-helix DNA-binding domain containing protein, expressed	GO:0007154	cell communication
LOC_Os11g40840.1	receptor-like protein kinase 2 precursor, putative, expressed	GO:0016049	cell growth
LOC_Os11g31090.1	transferase family protein, putative, expressed	GO:0016043	cellular component organization
LOC_Os11g32320.1	CCB1, putative, expressed	GO:0016043	cellular component organization
LOC_Os11g36390.1	RFC1 - Putative clamp loader of PCNA, replication factor C subunit 1, expressed	GO:0016043	cellular component organization
LOC_Os11g37100.1	expressed protein	GO:0016043	cellular component organization
LOC_Os11g38900.1	histone-lysine N-methyltransferase, H3 lysine-9 specific SUVH1, putative, expressed	GO:0016043	cellular component organization
LOC_Os11g40840.1	receptor-like protein kinase 2 precursor, putative, expressed	GO:0016043	cellular component organization
LOC_Os11g38040.1	expressed protein	GO:0019725	cellular homeostasis
LOC_Os11g31090.1	transferase family protein, putative, expressed	GO:0009987	cellular process
LOC_Os11g32320.1	CCB1, putative, expressed	GO:0009987	cellular process
LOC_Os11g36050.1	prefoldin subunit, putative, expressed	GO:0009987	cellular process
LOC_Os11g36390.1	RFC1 - Putative clamp loader of PCNA, replication factor C subunit 1, expressed	GO:0009987	cellular process

LOC_Os11g37000.1	heat shock protein DnaJ, putative, expressed	GO:0009987	cellular process
LOC_Os11g37130.1	mttA/Hcf106 family protein, putative, expressed	GO:0009987	cellular process
LOC_Os11g37140.1	expressed protein	GO:0009987	cellular process
LOC_Os11g37330.2	pentatricopeptide repeat domain containing protein, putative, expressed	GO:0009987	cellular process
LOC_Os11g37330.2	pentatricopeptide repeat domain containing protein, putative, expressed	GO:0009987	cellular process
LOC_Os11g37700.1	pleiotropic drug resistance protein, putative, expressed	GO:0009987	cellular process
LOC_Os11g37890.1	NAD dependent epimerase/dehydratase family protein, putative, expressed	GO:0009987	cellular process
LOC_Os11g37890.1	NAD dependent epimerase/dehydratase family protein, putative, expressed	GO:0009987	cellular process
LOC_Os11g37890.1	NAD dependent epimerase/dehydratase family protein, putative, expressed	GO:0009987	cellular process
LOC_Os11g38050.1	phosphoesterase family protein, putative, expressed	GO:0009987	cellular process
LOC_Os11g38620.1	expressed protein	GO:0009987	cellular process
LOC_Os11g38620.1	expressed protein	GO:0009987	cellular process
LOC_Os11g38630.1	expressed protein	GO:0009987	cellular process
LOC_Os11g38640.1	expressed protein	GO:0009987	cellular process
LOC_Os11g39650.1	WD domain, G-beta repeat domain containing protein, expressed	GO:0009987	cellular process
LOC_Os11g40840.1	receptor-like protein kinase 2 precursor, putative, expressed	GO:0009987	cellular process
LOC_Os11g30790.1	expressed protein	GO:0005575	cellular_component
LOC_Os11g31090.1	transferase family protein, putative, expressed	GO:0005575	cellular_component
LOC_Os11g37000.1	heat shock protein DnaJ, putative, expressed	GO:0005575	cellular_component
LOC_Os11g37100.1	expressed protein	GO:0005575	cellular_component
LOC_Os11g37300.1	OsFBDUF53 - F-box and DUF domain containing protein, expressed	GO:0005575	cellular_component
LOC_Os11g37870.1	stripe rust resistance protein Yr10, putative, expressed	GO:0005575	cellular_component
LOC_Os11g38010.1	targeting protein for Xklp2, putative, expressed	GO:0005575	cellular_component
LOC_Os11g38140.1	OsFBDUF58 - F-box and DUF domain containing protein, expressed	GO:0005575	cellular_component
LOC_Os11g39540.1	14-3-3 protein, putative, expressed	GO:0005575	cellular_component

LOC_Os11g40200.1	expressed protein	GO:0005575	cellular_component
LOC_Os11g40590.3	DUF1399 containing protein, putative, expressed	GO:0005575	cellular_component
LOC_Os11g40590.3	DUF1399 containing protein, putative, expressed	GO:0005575	cellular_component
LOC_Os11g40590.3	DUF1399 containing protein, putative, expressed	GO:0005575	cellular_component
LOC_Os11g37090.1	pumilio-family RNA binding repeat domain containing protein, expressed	GO:0005737	cytoplasm
LOC_Os11g38800.1	zinc finger family protein, putative, expressed	GO:0005737	cytoplasm
LOC_Os11g38800.1	zinc finger family protein, putative, expressed	GO:0005737	cytoplasm
LOC_Os11g39540.1	14-3-3 protein, putative, expressed	GO:0005737	cytoplasm
LOC_Os11g36050.1	prefoldin subunit, putative, expressed	GO:0005829	cytosol
LOC_Os11g36060.1	THUMP domain-containing protein, putative, expressed	GO:0005829	cytosol
LOC_Os11g36060.1	THUMP domain-containing protein, putative, expressed	GO:0005829	cytosol
LOC_Os11g37890.1	NAD dependent epimerase/dehydratase family protein, putative, expressed	GO:0005829	cytosol
LOC_Os11g37890.1	NAD dependent epimerase/dehydratase family protein, putative, expressed	GO:0005829	cytosol
LOC_Os11g37890.1	NAD dependent epimerase/dehydratase family protein, putative, expressed	GO:0005829	cytosol
LOC_Os11g39540.1	14-3-3 protein, putative, expressed	GO:0005829	cytosol
LOC_Os11g30370.1	OsSPL19 - SBP-box gene family member, expressed	GO:0003677	DNA binding
LOC_Os11g31500.1	ATP binding protein, putative, expressed	GO:0003677	DNA binding
LOC_Os11g36390.1	RFC1 - Putative clamp loader of PCNA, replication factor C subunit 1, expressed	GO:0003677	DNA binding
LOC_Os11g38870.1	helix-loop-helix DNA-binding domain containing protein, expressed	GO:0003677	DNA binding
LOC_Os11g38870.1	helix-loop-helix DNA-binding domain containing protein, expressed	GO:0003677	DNA binding
LOC_Os11g38810.1	mannose-6-phosphate isomerase, putative, expressed	GO:0009790	embryo development
LOC_Os11g38810.1	mannose-6-phosphate isomerase, putative, expressed	GO:0009790	embryo development
LOC_Os11g37260.1	SEY1, putative, expressed	GO:0005783	endoplasmic reticulum
LOC_Os11g39650.1	WD domain, G-beta repeat domain containing protein, expressed	GO:0005783	endoplasmic reticulum
LOC_Os11g37690.1	TBC domain containing protein, expressed	GO:0030234	enzyme regulator activity

LOC_Os11g30370.1	OsSPL19 - SBP-box gene family member, expressed	GO:0009908	flower development
LOC_Os11g31620.1	OsFBL55 - F-box domain and LRR containing protein, expressed	GO:0009908	flower development
LOC_Os11g36390.1	RFC1 - Putative clamp loader of PCNA, replication factor C subunit 1, expressed	GO:0016787	hydrolase activity
LOC_Os11g37700.1	pleiotropic drug resistance protein, putative, expressed	GO:0016787	hydrolase activity
LOC_Os11g38050.1	phosphoesterase family protein, putative, expressed	GO:0016787	hydrolase activity
LOC_Os11g38620.1	expressed protein	GO:0016787	hydrolase activity
LOC_Os11g38620.1	expressed protein	GO:0016787	hydrolase activity
LOC_Os11g38630.1	expressed protein	GO:0016787	hydrolase activity
LOC_Os11g38640.1	expressed protein	GO:0016787	hydrolase activity
LOC_Os11g31650.2	expressed protein	GO:0005622	intracellular
LOC_Os11g31650.2	expressed protein	GO:0005622	intracellular
LOC_Os11g36390.1	RFC1 - Putative clamp loader of PCNA, replication factor C subunit 1, expressed	GO:0005622	intracellular
LOC_Os11g37510.1	ribosomal protein L4, putative, expressed	GO:0005622	intracellular
LOC_Os11g38620.1	expressed protein	GO:0005622	intracellular
LOC_Os11g38620.1	expressed protein	GO:0005622	intracellular
LOC_Os11g38630.1	expressed protein	GO:0005622	intracellular
LOC_Os11g38640.1	expressed protein	GO:0005622	intracellular
LOC_Os11g36090.1	receptor kinase, putative, expressed	GO:0016301	kinase activity
LOC_Os11g36140.1	receptor-like protein kinase 2 precursor, putative, expressed	GO:0016301	kinase activity
LOC_Os11g36180.1	receptor kinase, putative, expressed	GO:0016301	kinase activity
LOC_Os11g40840.1	receptor-like protein kinase 2 precursor, putative, expressed	GO:0016301	kinase activity
LOC_Os11g30560.1	dehydrogenase/reductase, putative, expressed	GO:0006629	lipid metabolic process
LOC_Os11g31090.1	transferase family protein, putative, expressed	GO:0006629	lipid metabolic process
LOC_Os11g37330.2	pentatricopeptide repeat domain containing protein, putative, expressed	GO:0006629	lipid metabolic process
LOC_Os11g37330.2	pentatricopeptide repeat domain containing protein, putative, expressed	GO:0006629	lipid metabolic process
LOC_Os11g38050.1	phosphoesterase family protein, putative, expressed	GO:0006629	lipid metabolic process
LOC_Os11g13750.1	expressed protein	GO:0016020	membrane

LOC_Os11g31620.1	OsFBL55 - F-box domain and LRR containing protein, expressed	GO:0016020	membrane
LOC_Os11g37130.1	mttA/Hcf106 family protein, putative, expressed	GO:0016020	membrane
LOC_Os11g37700.1	pleiotropic drug resistance protein, putative, expressed	GO:0016020	membrane
LOC_Os11g39650.1	WD domain, G-beta repeat domain containing protein, expressed	GO:0016020	membrane
LOC_Os11g30560.1	dehydrogenase/reductase, putative, expressed	GO:0008152	metabolic process
LOC_Os11g31090.1	transferase family protein, putative, expressed	GO:0008152	metabolic process
LOC_Os11g36390.1	RFC1 - Putative clamp loader of PCNA, replication factor C subunit 1, expressed	GO:0008152	metabolic process
LOC_Os11g37140.1	expressed protein	GO:0008152	metabolic process
LOC_Os11g37330.2	pentatricopeptide repeat domain containing protein, putative, expressed	GO:0008152	metabolic process
LOC_Os11g37330.2	pentatricopeptide repeat domain containing protein, putative, expressed	GO:0008152	metabolic process
LOC_Os11g37890.1	NAD dependent epimerase/dehydratase family protein, putative, expressed	GO:0008152	metabolic process
LOC_Os11g37890.1	NAD dependent epimerase/dehydratase family protein, putative, expressed	GO:0008152	metabolic process
LOC_Os11g37890.1	NAD dependent epimerase/dehydratase family protein, putative, expressed	GO:0008152	metabolic process
LOC_Os11g38050.1	phosphoesterase family protein, putative, expressed	GO:0008152	metabolic process
LOC_Os11g38810.1	mannose-6-phosphate isomerase, putative, expressed	GO:0008152	metabolic process
LOC_Os11g38810.1	mannose-6-phosphate isomerase, putative, expressed	GO:0008152	metabolic process
LOC_Os11g36070.3	expressed protein	GO:0005739	mitochondrion
LOC_Os11g36070.3	expressed protein	GO:0005739	mitochondrion
LOC_Os11g36070.3	expressed protein	GO:0005739	mitochondrion
LOC_Os11g36070.3	expressed protein	GO:0005739	mitochondrion
LOC_Os11g36070.3	expressed protein	GO:0005739	mitochondrion
LOC_Os11g36070.3	expressed protein	GO:0005739	mitochondrion
LOC_Os11g36070.3	expressed protein	GO:0005739	mitochondrion
LOC_Os11g37330.2	pentatricopeptide repeat domain containing protein, putative, expressed	GO:0005739	mitochondrion
LOC_Os11g37330.2	pentatricopeptide repeat domain containing protein, putative, expressed	GO:0005739	mitochondrion

LOC_Os11g37690.1	TBC domain containing protein, expressed	GO:0005739	mitochondrion
LOC_Os11g39540.1	14-3-3 protein, putative, expressed	GO:0005739	mitochondrion
LOC_Os11g40840.1	receptor-like protein kinase 2 precursor, putative, expressed	GO:0005739	mitochondrion
LOC_Os11g32320.1	CCB1, putative, expressed	GO:0003674	molecular_function
LOC_Os11g32360.1	expressed protein	GO:0003674	molecular_function
LOC_Os11g32360.1	expressed protein	GO:0003674	molecular_function
LOC_Os11g36060.1	THUMP domain-containing protein, putative, expressed	GO:0003674	molecular_function
LOC_Os11g36060.1	THUMP domain-containing protein, putative, expressed	GO:0003674	molecular_function
LOC_Os11g36070.3	expressed protein	GO:0003674	molecular_function
LOC_Os11g36070.3	expressed protein	GO:0003674	molecular_function
LOC_Os11g36070.3	expressed protein	GO:0003674	molecular_function
LOC_Os11g36070.3	expressed protein	GO:0003674	molecular_function
LOC_Os11g36070.3	expressed protein	GO:0003674	molecular_function
LOC_Os11g36070.3	expressed protein	GO:0003674	molecular_function
LOC_Os11g36070.3	expressed protein	GO:0003674	molecular_function
LOC_Os11g36340.1	lymphoid organ expressed yellow head virus receptor protein, putative, expressed	GO:0003674	molecular_function
LOC_Os11g36350.1	OsFBDUF50 - F-box and DUF domain containing protein, expressed	GO:0003674	molecular_function
LOC_Os11g37300.1	OsFBDUF53 - F-box and DUF domain containing protein, expressed	GO:0003674	molecular_function
LOC_Os11g38010.1	targeting protein for Xklp2, putative, expressed	GO:0003674	molecular_function
LOC_Os11g38140.1	OsFBDUF58 - F-box and DUF domain containing protein, expressed	GO:0003674	molecular_function
LOC_Os11g37140.1	expressed protein	GO:0003774	motor activity
LOC_Os11g31620.1	OsFBL55 - F-box domain and LRR containing protein, expressed	GO:0007275	multicellular organismal development
LOC_Os11g37330.2	pentatricopeptide repeat domain containing protein, putative, expressed	GO:0007275	multicellular organismal development
LOC_Os11g37330.2	pentatricopeptide repeat domain containing protein, putative, expressed	GO:0007275	multicellular organismal development
LOC_Os11g40840.1	receptor-like protein kinase 2 precursor, putative, expressed	GO:0007275	multicellular organismal development

LOC_Os11g30370.1	OsSPL19 - SBP-box gene family member, expressed	GO:0006139	nucleobase, nucleoside, nucleotide and nucleic acid metabolic process
LOC_Os11g36390.1	RFC1 - Putative clamp loader of PCNA, replication factor C subunit 1, expressed	GO:0006139	nucleobase, nucleoside, nucleotide and nucleic acid metabolic process
LOC_Os11g37100.1	expressed protein	GO:0006139	nucleobase, nucleoside, nucleotide and nucleic acid metabolic process
LOC_Os11g37690.1	TBC domain containing protein, expressed	GO:0006139	nucleobase, nucleoside, nucleotide and nucleic acid metabolic process
LOC_Os11g37700.1	pleiotropic drug resistance protein, putative, expressed	GO:0006139	nucleobase, nucleoside, nucleotide and nucleic acid metabolic process
LOC_Os11g37890.1	NAD dependent epimerase/dehydratase family protein, putative, expressed	GO:0006139	nucleobase, nucleoside, nucleotide and nucleic acid metabolic process
LOC_Os11g37890.1	NAD dependent epimerase/dehydratase family protein, putative, expressed	GO:0006139	nucleobase, nucleoside, nucleotide and nucleic acid metabolic process
LOC_Os11g37890.1	NAD dependent epimerase/dehydratase family protein, putative, expressed	GO:0006139	nucleobase, nucleoside, nucleotide and nucleic acid metabolic process
LOC_Os11g38870.1	helix-loop-helix DNA-binding domain containing protein, expressed	GO:0006139	nucleobase, nucleoside, nucleotide and nucleic acid metabolic process
LOC_Os11g38870.1	helix-loop-helix DNA-binding domain containing protein, expressed	GO:0006139	nucleobase, nucleoside, nucleotide and nucleic acid metabolic process
LOC_Os11g31500.1	ATP binding protein, putative, expressed	GO:0000166	nucleotide binding
LOC_Os11g32210.1	jacalin-like lectin domain containing protein, expressed	GO:0000166	nucleotide binding
LOC_Os11g36090.1	receptor kinase, putative, expressed	GO:0000166	nucleotide binding
LOC_Os11g36140.1	receptor-like protein kinase 2 precursor, putative, expressed	GO:0000166	nucleotide binding
LOC_Os11g36180.1	receptor kinase, putative, expressed	GO:0000166	nucleotide binding
LOC_Os11g36390.1	RFC1 - Putative clamp loader of PCNA, replication factor C subunit 1, expressed	GO:0000166	nucleotide binding
LOC_Os11g37140.1	expressed protein	GO:0000166	nucleotide binding
LOC_Os11g37260.1	SEY1, putative, expressed	GO:0000166	nucleotide binding
LOC_Os11g37860.1	stripe rust resistance protein Yr10, putative, expressed	GO:0000166	nucleotide binding
LOC_Os11g37870.1	stripe rust resistance protein Yr10, putative, expressed	GO:0000166	nucleotide binding
LOC_Os11g37890.1	NAD dependent epimerase/dehydratase family protein, putative, expressed	GO:0000166	nucleotide binding
LOC_Os11g37890.1	NAD dependent epimerase/dehydratase family protein, putative, expressed	GO:0000166	nucleotide binding

LOC_Os11g37890.1	NAD dependent epimerase/dehydratase family protein, putative, expressed	GO:0000166	nucleotide binding
LOC_Os11g38020.1	GTPase of unknown function domain containing protein, putative, expressed	GO:0000166	nucleotide binding
LOC_Os11g39540.1	14-3-3 protein, putative, expressed	GO:0000166	nucleotide binding
LOC_Os11g39650.1	WD domain, G-beta repeat domain containing protein, expressed	GO:0000166	nucleotide binding
LOC_Os11g30370.1	OsSPL19 - SBP-box gene family member, expressed	GO:0005634	nucleus
LOC_Os11g37090.1	pumilio-family RNA binding repeat domain containing protein, expressed	GO:0005634	nucleus
LOC_Os11g38800.1	zinc finger family protein, putative, expressed	GO:0005634	nucleus
LOC_Os11g38800.1	zinc finger family protein, putative, expressed	GO:0005634	nucleus
LOC_Os11g38870.1	helix-loop-helix DNA-binding domain containing protein, expressed	GO:0005634	nucleus
LOC_Os11g38870.1	helix-loop-helix DNA-binding domain containing protein, expressed	GO:0005634	nucleus
LOC_Os11g38900.1	histone-lysine N-methyltransferase, H3 lysine-9 specific SUVH1, putative, expressed	GO:0005634	nucleus
LOC_Os11g31950.1	expressed protein	GO:0005886	plasma membrane
LOC_Os11g36090.1	receptor kinase, putative, expressed	GO:0005886	plasma membrane
LOC_Os11g36140.1	receptor-like protein kinase 2 precursor, putative, expressed	GO:0005886	plasma membrane
LOC_Os11g36180.1	receptor kinase, putative, expressed	GO:0005886	plasma membrane
LOC_Os11g38050.1	phosphoesterase family protein, putative, expressed	GO:0005886	plasma membrane
LOC_Os11g39540.1	14-3-3 protein, putative, expressed	GO:0005886	plasma membrane
LOC_Os11g39650.1	WD domain, G-beta repeat domain containing protein, expressed	GO:0005886	plasma membrane
LOC_Os11g31500.1	ATP binding protein, putative, expressed	GO:0009536	plastid
LOC_Os11g32320.1	CCB1, putative, expressed	GO:0009536	plastid
LOC_Os11g37130.1	mttA/Hcf106 family protein, putative, expressed	GO:0009536	plastid
LOC_Os11g37140.1	expressed protein	GO:0009536	plastid
LOC_Os11g37510.1	ribosomal protein L4, putative, expressed	GO:0009536	plastid
LOC_Os11g38020.1	GTPase of unknown function domain containing protein, putative, expressed	GO:0009536	plastid
LOC_Os11g38870.1	helix-loop-helix DNA-binding domain containing protein, expressed	GO:0009536	plastid

LOC_Os11g38870.1	helix-loop-helix DNA-binding domain containing protein, expressed	GO:0009536	plastid
LOC_Os11g39540.1	14-3-3 protein, putative, expressed	GO:0009536	plastid
LOC_Os11g38810.1	mannose-6-phosphate isomerase, putative, expressed	GO:0009791	post-embryonic developmen
LOC_Os11g38810.1	mannose-6-phosphate isomerase, putative, expressed	GO:0009791	post-embryonic developmen
LOC_Os11g40840.1	receptor-like protein kinase 2 precursor, putative, expressed	GO:0009791	post-embryonic developmen
LOC_Os11g31620.1	OsFBL55 - F-box domain and LRR containing protein, expressed	GO:0005515	protein binding
LOC_Os11g36050.1	prefoldin subunit, putative, expressed	GO:0005515	protein binding
LOC_Os11g37000.1	heat shock protein DnaJ, putative, expressed	GO:0005515	protein binding
LOC_Os11g37960.1	WIP4 - Wound-induced protein precursor, expressed	GO:0005515	protein binding
LOC_Os11g39540.1	14-3-3 protein, putative, expressed	GO:0005515	protein binding
LOC_Os11g45620.1	rust-resistance protein Lr21, putative, expressed	GO:0005515	protein binding
LOC_Os11g36050.1	prefoldin subunit, putative, expressed	GO:0019538	protein metabolic process
LOC_Os11g37000.1	heat shock protein DnaJ, putative, expressed	GO:0019538	protein metabolic process
LOC_Os11g38620.1	expressed protein	GO:0019538	protein metabolic process
LOC_Os11g38620.1	expressed protein	GO:0019538	protein metabolic process
LOC_Os11g38630.1	expressed protein	GO:0019538	protein metabolic process
LOC_Os11g38640.1	expressed protein	GO:0019538	protein metabolic process
LOC_Os11g31620.1	OsFBL55 - F-box domain and LRR containing protein, expressed	GO:0006464	protein modification process
LOC_Os11g36090.1	receptor kinase, putative, expressed	GO:0006464	protein modification process
LOC_Os11g36140.1	receptor-like protein kinase 2 precursor, putative, expressed	GO:0006464	protein modification process
LOC_Os11g36180.1	receptor kinase, putative, expressed	GO:0006464	protein modification process
LOC_Os11g36390.1	RFC1 - Putative clamp loader of PCNA, replication factor C subunit 1, expressed	GO:0006464	protein modification process
LOC_Os11g38900.1	histone-lysine N-methyltransferase, H3 lysine-9 specific SUVH1, putative, expressed	GO:0006464	protein modification process
LOC_Os11g40840.1	receptor-like protein kinase 2 precursor, putative, expressed	GO:0006464	protein modification process
LOC_Os11g40840.1	receptor-like protein kinase 2 precursor, putative, expressed	GO:0004872	receptor activity
LOC_Os11g36390.1	RFC1 - Putative clamp loader of PCNA, replication factor C subunit 1, expressed	GO:0040029	regulation of gene expression, epigenetic

LOC_Os11g38900.1	histone-lysine N-methyltransferase, H3 lysine-9 specific SUVH1, putative, expressed	GO:0040029	regulation of gene expression, epigenetic
LOC_Os11g38810.1	mannose-6-phosphate isomerase, putative, expressed	GO:0000003	reproduction
LOC_Os11g38810.1	mannose-6-phosphate isomerase, putative, expressed	GO:0000003	reproduction
LOC_Os11g40840.1	receptor-like protein kinase 2 precursor, putative, expressed	GO:0000003	reproduction
LOC_Os11g37960.1	WIP4 - Wound-induced protein precursor, expressed	GO:0009628	response to abiotic stimulus
LOC_Os11g31620.1	OsFBL55 - F-box domain and LRR containing protein, expressed	GO:0009607	response to biotic stimulus
LOC_Os11g37960.1	WIP4 - Wound-induced protein precursor, expressed	GO:0009607	response to biotic stimulus
LOC_Os11g40840.1	receptor-like protein kinase 2 precursor, putative, expressed	GO:0009607	response to biotic stimulus
LOC_Os11g38870.1	helix-loop-helix DNA-binding domain containing protein, expressed	GO:0009991	response to extracellular stimulus
LOC_Os11g38870.1	helix-loop-helix DNA-binding domain containing protein, expressed	GO:0009991	response to extracellular stimulus
LOC_Os11g32210.1	jacalin-like lectin domain containing protein, expressed	GO:0006950	response to stress
LOC_Os11g36390.1	RFC1 - Putative clamp loader of PCNA, replication factor C subunit 1, expressed	GO:0006950	response to stress
LOC_Os11g37860.1	stripe rust resistance protein Yr10, putative, expressed	GO:0006950	response to stress
LOC_Os11g37870.1	stripe rust resistance protein Yr10, putative, expressed	GO:0006950	response to stress
LOC_Os11g37960.1	WIP4 - Wound-induced protein precursor, expressed	GO:0006950	response to stress
LOC_Os11g38870.1	helix-loop-helix DNA-binding domain containing protein, expressed	GO:0006950	response to stress
LOC_Os11g38870.1	helix-loop-helix DNA-binding domain containing protein, expressed	GO:0006950	response to stress
LOC_Os11g40840.1	receptor-like protein kinase 2 precursor, putative, expressed	GO:0006950	response to stress
LOC_Os11g45620.1	rust-resistance protein Lr21, putative, expressed	GO:0006950	response to stress
LOC_Os11g31650.2	expressed protein	GO:0005840	ribosome
LOC_Os11g31650.2	expressed protein	GO:0005840	ribosome
LOC_Os11g37510.1	ribosomal protein L4, putative, expressed	GO:0005840	ribosome
LOC_Os11g37090.1	pumilio-family RNA binding repeat domain containing protein, expressed	GO:0003723	RNA binding
LOC_Os11g37330.2	pentatricopeptide repeat domain containing protein, putative, expressed	GO:0003723	RNA binding

LOC_Os11g37330.2	pentatricopeptide repeat domain containing protein, putative, expressed	GO:0003723	RNA binding
LOC_Os11g37510.1	ribosomal protein L4, putative, expressed	GO:0003723	RNA binding
LOC_Os11g31090.1	transferase family protein, putative, expressed	GO:0019748	secondary metabolic process
LOC_Os11g30370.1	OsSPL19 - SBP-box gene family member, expressed	GO:0003700	sequence-specific DNA binding transcription factor activity
LOC_Os11g38870.1	helix-loop-helix DNA-binding domain containing protein, expressed	GO:0003700	sequence-specific DNA binding transcription factor activity
LOC_Os11g38870.1	helix-loop-helix DNA-binding domain containing protein, expressed	GO:0003700	sequence-specific DNA binding transcription factor activity
LOC_Os11g40840.1	receptor-like protein kinase 2 precursor, putative, expressed	GO:0004871	signal transducer activity
LOC_Os11g36090.1	receptor kinase, putative, expressed	GO:0007165	signal transduction
LOC_Os11g36140.1	receptor-like protein kinase 2 precursor, putative, expressed	GO:0007165	signal transduction
LOC_Os11g36180.1	receptor kinase, putative, expressed	GO:0007165	signal transduction
LOC_Os11g37690.1	TBC domain containing protein, expressed	GO:0007165	signal transduction
LOC_Os11g39540.1	14-3-3 protein, putative, expressed	GO:0007165	signal transduction
LOC_Os11g40840.1	receptor-like protein kinase 2 precursor, putative, expressed	GO:0007165	signal transduction
LOC_Os11g31650.2	expressed protein	GO:0005198	structural molecule activity
LOC_Os11g31650.2	expressed protein	GO:0005198	structural molecule activity
LOC_Os11g37510.1	ribosomal protein L4, putative, expressed	GO:0005198	structural molecule activity
LOC_Os11g37130.1	mttA/Hcf106 family protein, putative, expressed	GO:0009579	thylakoid
LOC_Os11g31090.1	transferase family protein, putative, expressed	GO:0016740	transferase activity
LOC_Os11g38900.1	histone-lysine N-methyltransferase, H3 lysine-9 specific SUVH1, putative, expressed	GO:0016740	transferase activity
LOC_Os11g31650.2	expressed protein	GO:0006412	translation
LOC_Os11g31650.2	expressed protein	GO:0006412	translation
LOC_Os11g37510.1	ribosomal protein L4, putative, expressed	GO:0006412	translation
LOC_Os11g37130.1	mttA/Hcf106 family protein, putative, expressed	GO:0006810	transport
LOC_Os11g37700.1	pleiotropic drug resistance protein, putative, expressed	GO:0006810	transport
LOC_Os11g39650.1	WD domain, G-beta repeat domain containing protein, expressed	GO:0006810	transport
LOC_Os11g40840.1	receptor-like protein kinase 2 precursor, putative, expressed	GO:0006810	transport

LOC_Os11g37130.1	mttA/Hcf106 family protein, putative, expressed	GO:0005215	transporter activity
LOC_Os11g37700.1	pleiotropic drug resistance protein, putative, expressed	GO:0005215	transporter activity
LOC_Os11g13750.1	expressed protein	GO:0005773	vacuole
LOC_Os11g31620.1	OsFBL55 - F-box domain and LRR containing protein, expressed	GO:0005773	vacuole
LOC_Os11g39650.1	WD domain, G-beta repeat domain containing protein, expressed	GO:0005773	vacuole

Table S4. snpEff results indicating potential influence of identified SNPs outside of linkage block influence of known *Xa* genes. Strongest potential effect, 'moderate', is shown in underlined bold font.

<u>CHROMOSOME 5</u>

LOC_Os05g05700 2838372 R05002838372 A G . . PR;ANN=
G|3_prime_UTR_variant|MODIFIER|OS05G0149600|OS05G0149600|transcript|OS05T0149600-
01|protein_coding|5/18|c.*302T>C||||457|WARNING_REF_DOES_NOT_MATCH_GENOME
G|upstream_gene_variant|MODIFIER|OS05G0149600|OS05G0149600|transcript|OS05T0149600
02|protein_coding||c.-2344T>C||||2342|WARNING_TRANSCRIPT_NO_START_CODON
G|downstream_gene_variant|MODIFIER|OS05G0149500|OS05G0149500|transcript|OS05T0149
00-01|protein_coding||c.*4761A>G||||4608|WARNING_TRANSCRIPT_NO_START_CODON

LOC_Os05g05700 2840837 R05002840837 G A . . PR;ANN=
A|5_prime_UTR_premature_start_codon_gain_variant|LOW|OS05G0149600|OS05G0149600|tra
nscript|OS05T0149600-01|protein_coding|1/18|c.-
180C>T|||||WARNING_REF_DOES_NOT_MATCH_GENOME
A|5_prime_UTR_variant|MODIFIER|OS05G0149600|OS05G0149600|transcript|OS05T0149600-
01|protein_coding|1/18|c.-180C>T||||180|WARNING_REF_DOES_NOT_MATCH_GENOME
A|upstream_gene_variant|MODIFIER|OS05G0149600|OS05G0149600|transcript|OS05T0149600
02|protein_coding||c.-4809C>T||||4807|WARNING_TRANSCRIPT_NO_START_CODON
A|upstream_gene_variant|MODIFIER|EPlOSAG00000039380|EPlOSAG00000039380|transcript
EPlOSAT00000040768|lincRNA||n.-3879G>A||||3879|
A|upstream_gene_variant|MODIFIER|OS05G0149800|OS05G0149800|transcript|OS05T0149800
01|protein_coding||c.-4970G>A||||4859|
A|downstream_gene_variant|MODIFIER|EPlOSAG00000036853|EPlOSAG00000036853|transcr
pt|EPlOSAT00000038241|lincRNA||n.*3879C>T||||3879|
A|downstream_gene_variant|MODIFIER|EPlOSAG00000001261|EPlOSAG00000001261|transcr
pt|EPlOSAT00000002649|lincRNA||n.*3901C>T||||3901|
A|downstream_gene_variant|MODIFIER|OS05G0149701|OS05G0149701|transcript|OS05T0149
01-00|protein_coding||c.*6393C>T||||4969|

LOC_Os05g05800 2895375 R05002895375 T G . . PR;ANN=
G|intron_variant|MODIFIER|OS05G0150500|OS05G0150500|transcript|OS05T0150500-
00|protein_coding|1/2|c.501-154A>C|||||
G|intron_variant|MODIFIER|OS05G0150550|OS05G0150550|transcript|OS05T0150550-
00|protein_coding|2/2|c.-46+168T>G|||||

<u>CHROMOSOME 11</u>

LOC_Os11g32210 19023042 R11019023042 T C . .
PR;ANN=C|intron_variant|MODIFIER|OS11G0524900|OS11G0524900|transcript|OS11T05
24900-00|protein_coding|6/7|c.1885-39A>G|||||

LOC_Os11g32210 19024056 R11019024056 G A . .
PR;ANN=A|intron_variant|MODIFIER|OS11G0524900|OS11G0524900|transcript|OS11T05
24900-00|protein_coding|4/7|c.1558-87C>T|||||

LOC_Os11g32210 **19025182*** R11019025182 C A . .
PR;ANN=A|synonymous_variant|LOW|OS11G0524900|OS11G0524900|transcript|OS11T0
524900-00|protein_coding|3/8|c.1251G>T|p.Val417Val|1251/2121|1251/2121|417/706||

<table>
<tr><td>LOC_Os11g32210</td><td>19026330</td><td>R11019026330</td><td>C</td><td>G</td><td>.</td><td>.</td></tr>
<tr><td colspan="7">PR;ANN=G|missense_variant|MODERATE|OS11G0524900|OS11G0524900|transcript|OS
1T0524900-00|protein_coding|1/8|c.291G>C|p.Lys97Asn|291/2121|291/2121|97/706||,
G|downstream_gene_variant|MODIFIER|EPlOSAG00000051154|EPlOSAG00000051154|transcr
pt|EPlOSAT00000052542|lincRNA||n.*4063G>C||||4063|</td></tr>
<tr><td>LOC_Os11g32210</td><td>19028382</td><td>R11019028382</td><td>A</td><td>T</td><td>.</td><td>.</td></tr>
<tr><td colspan="7">PR;ANN=T|upstream_gene_variant|MODIFIER|OS11G0524900|OS11G0524900|transcript
OS11T0524900-00|protein_coding||c.-1762T>A||||1762|,
T|downstream_gene_variant|MODIFIER|EPlOSAG00000051154|EPlOSAG00000051154|transcri
t|EPlOSAT00000052542|lincRNA||n.*2011T>A||||2011|,
T|intergenic_region|MODIFIER|OS11G0524900-EPlOSAG00000051154|OS11G0524900-
EPlOSAG00000051154|intergenic_region|OS11G0524900-
EPlOSAG00000051154|||n.19028382A>T||||||</td></tr>
<tr><td>LOC_Os11g36390</td><td>21443514</td><td>R11021443514</td><td>G</td><td>C</td><td>.</td><td>.</td></tr>
<tr><td colspan="7">PR;ANN=C|upstream_gene_variant|MODIFIER|RFC1|OS11G0572100|transcript|OS11T05
72100-03|protein_coding||c.-
1458G>C||||1456|WARNING_TRANSCRIPT_NO_START_CODON,
C|intron_variant|MODIFIER|RFC1|OS11G0572100|transcript|OS11T0572100-
01|protein_coding|1/21|c.3+58G>C||||||,
C|intron_variant|MODIFIER|RFC1|OS11G0572100|transcript|OS11T0572100-
02|protein_coding|1/21|c.3+58G>C||||||</td></tr>
<tr><td>LOC_Os11g36390</td><td>21444533*</td><td>R11021444533</td><td>G</td><td>C</td><td>.</td><td>.</td></tr>
<tr><td colspan="7">PR;ANN=C|synonymous_variant|LOW|RFC1|OS11G0572100|transcript|OS11T0572100-
01|protein_coding|3/22|c.387G>C|p.Val129Val|789/3747|387/3066|129/1021||,
C|synonymous_variant|LOW|RFC1|OS11G0572100|transcript|OS11T0572100-
02|protein_coding|3/22|c.387G>C|p.Val129Val|716/3675|387/3066|129/1021||,
C|upstream_gene_variant|MODIFIER|RFC1|OS11G0572100|transcript|OS11T0572100-
03|protein_coding||c.-
439G>C||||437|WARNING_TRANSCRIPT_NO_START_CODON,C|upstream_gene_variant|M
ODIFIER|RFC1|OS11G0572100|transcript|OS11T0572100-04|protein_coding||c.-
4626G>C||||4626|WARNING_TRANSCRIPT_NO_START_CODON</td></tr>
<tr><td>LOC_Os11g36390</td><td>21445643*</td><td>R11021445643</td><td>G</td><td>A</td><td>.</td><td>.</td></tr>
<tr><td colspan="7">PR;ANN=A|upstream_gene_variant|MODIFIER|RFC1|OS11G0572100|transcript|OS11T05
72100-04|protein_coding||c.-
3516G>A||||3516|WARNING_TRANSCRIPT_NO_START_CODON,
A|intron_variant|MODIFIER|RFC1|OS11G0572100|transcript|OS11T0572100-
01|protein_coding|7/21|c.1114+11G>A||||||,
A|intron_variant|MODIFIER|RFC1|OS11G0572100|transcript|OS11T0572100-
02|protein_coding|7/21|c.1114+11G>A||||||,
A|intron_variant|MODIFIER|RFC1|OS11G0572100|transcript|OS11T0572100-
03|protein_coding|4/18|c.358+11G>A||||||WARNING_TRANSCRIPT_NO_START_CODON</td></tr>
<tr><td>LOC_Os11g36390</td><td>21446864*</td><td>R11021446864</td><td>G</td><td>C</td><td>.</td><td>.</td></tr>
<tr><td colspan="7">PR;ANN=C|splice_region_variant&intron_variant|LOW|RFC1|OS11G0572100|transcript|
OS11T0572100-01|protein_coding|11/21|c.1797+8G>C||||||,
C|splice_region_variant&intron_variant|LOW|RFC1|OS11G0572100|transcript|OS11T0572100-
02|protein_coding|11/21|c.1797+8G>C||||||,
C|splice_region_variant&intron_variant|LOW|RFC1|OS11G0572100|transcript|OS11T0572100-
03|protein_coding|8/18|c.1041+8G>C||||||WARNING_TRANSCRIPT_NO_START_CODON,
C|upstream_gene_variant|MODIFIER|EPlOSAG00000038391|EPlOSAG00000038391|transcript|
EPlOSAT00000039779|lincRNA||n.-4972G>C||||4972|,
C|upstream_gene_variant|MODIFIER|EPlOSAG00000001127|EPlOSAG00000001127|transcript|
EPlOSAT00000002515|lincRNA||n.-4994G>C||||4994|,
C|upstream_gene_variant|MODIFIER|RFC1|OS11G0572100|transcript|OS11T0572100-
04|protein_coding||c.-2295G>C||||2295|WARNING_TRANSCRIPT_NO_START_CODON,
C|downstream_gene_variant|MODIFIER|EPlOSAG00000001435|EPlOSAG00000001435|transcr
pt|EPlOSAT00000002823|lincRNA||n.*4994C>G||||4994|,C|downstream_gene_variant|MOD</td></tr>
</table>

FIER|EPlOSAG00000037673|EPlOSAG00000037673|transcript|EPlOSAT00000039061|lincRNA|
n.*4998C>G|||||4998|

LOC_Os11g36390 **21451325*** R11021451325 G A . .
PR;ANN=A|upstream_gene_variant|MODIFIER|EPlOSAG00000038391|EPlOSAG0000003
391|transcript|EPlOSAT00000039779|lincRNA||n.-511G>A||||511|,
A|upstream_gene_variant|MODIFIER|EPlOSAG00000001127|EPlOSAG00000001127|transcript
EPlOSAT00000002515|lincRNA||n.-533G>A||||533|,
A|downstream_gene_variant|MODIFIER|EPlOSAG00000001435|EPlOSAG00000001435|transcr
pt|EPlOSAT00000002823|lincRNA||n.*533C>T|||||533|,
A|downstream_gene_variant|MODIFIER|EPlOSAG00000037673|EPlOSAG00000037673|transcr
pt|EPlOSAT00000039061|lincRNA||n.*537C>T|||||537|,
A|intron_variant|MODIFIER|RFC1|OS11G0572100|transcript|OS11T0572100-
01|protein_coding|19/21|c.2626-186G>A||||||,
A|intron_variant|MODIFIER|RFC1|OS11G0572100|transcript|OS11T0572100-
02|protein_coding|19/21|c.2626-186G>A||||||,
A|intron_variant|MODIFIER|RFC1|OS11G0572100|transcript|OS11T0572100-
03|protein_coding|16/18|c.1870-186G>A||||||WARNING_TRANSCRIPT_NO_START_CODON
A|intron_variant|MODIFIER|RFC1|OS11G0572100|transcript|OS11T0572100-
04|protein_coding|5/7|c.394-186G>A||||||WARNING_TRANSCRIPT_NO_START_CODON

LOC_Os11g36390 **21452470*** R11021452470 A G . .
PR;ANN=G|upstream_gene_variant|MODIFIER|EPlOSAG00000001435|EPlOSAG0000000
435|transcript|EPlOSAT00000002823|lincRNA||n.-495T>C|||||495|,
G|upstream_gene_variant|MODIFIER|EPlOSAG00000037673|EPlOSAG00000037673|transcript
EPlOSAT00000039061|lincRNA||n.-499T>C|||||499|,
G|downstream_gene_variant|MODIFIER|EPlOSAG00000038391|EPlOSAG00000038391|transcr
pt|EPlOSAT00000039779|lincRNA||n.*473A>G|||||473|,
G|downstream_gene_variant|MODIFIER|EPlOSAG00000001127|EPlOSAG00000001127|transcr
pt|EPlOSAT00000002515|lincRNA||n.*495A>G|||||495|,
G|intron_variant|MODIFIER|RFC1|OS11G0572100|transcript|OS11T0572100-
01|protein_coding|21/21|c.2933-20A>G||||||,
G|intron_variant|MODIFIER|RFC1|OS11G0572100|transcript|OS11T0572100-
02|protein_coding|21/21|c.2933-20A>G||||||,
G|intron_variant|MODIFIER|RFC1|OS11G0572100|transcript|OS11T0572100-
03|protein_coding|18/18|c.2177-20A>G||||||WARNING_TRANSCRIPT_NO_START_CODON
G|intron_variant|MODIFIER|RFC1|OS11G0572100|transcript|OS11T0572100-
04|protein_coding|7/7|c.701-20A>G||||||WARNING_TRANSCRIPT_NO_START_CODON

LOC_Os11g37740 **22311354*** R11022311354 A G . .
PR;ANN=G|upstream_gene_variant|MODIFIER|EPlOSAG00000036068|EPlOSAG0000003
068|transcript|EPlOSAT00000037456|lincRNA||n.-2771T>C|||||2771|,
G|downstream_gene_variant|MODIFIER|OS11G0588300|OS11G0588300|transcript|OS11T0588
00-01|protein_coding||c.*1556A>G|||||1273|,
G|downstream_gene_variant|MODIFIER|EPlOSAG00000036595|EPlOSAG00000036595|transcr
pt|EPlOSAT00000037983|lincRNA||n.*2771A>G|||||2771|,
G|downstream_gene_variant|MODIFIER|OS11G0588300|OS11G0588300|transcript|OS11T0588
00-02|protein_coding||c.*1556A>G|||||1273|,
G|intron_variant|MODIFIER|OS11G0588400|OS11G0588400|transcript|OS11T0588400-
01|protein_coding|1/4|c.-98+209A>G||||||

LOC_Os11g37740 **22312430*** R11022312430 G T . .
PR;ANN=T|upstream_gene_variant|MODIFIER|EPlOSAG00000036068|EPlOSAG0000003
68|transcript|EPlOSAT00000037456|lincRNA||n.-3847C>A|||||3847|,
T|downstream_gene_variant|MODIFIER|OS11G0588300|OS11G0588300|transcript|OS11T0588
00-01|protein_coding||c.*2632G>T|||||2349|,
T|downstream_gene_variant|MODIFIER|EPlOSAG00000036595|EPlOSAG00000036595|transcri
t|EPlOSAT00000037983|lincRNA||n.*3847G>T|||||3847|,
T|downstream_gene_variant|MODIFIER|OS11G0588300|OS11G0588300|transcript|OS11T0588
00-02|protein_coding||c.*2632G>T|||||2349|,
T|intron_variant|MODIFIER|OS11G0588400|OS11G0588400|transcript|OS11T0588400-
01|protein_coding|1/4|c.-98+1285G>T||||||

**LOC_Os11g37740**	22314880	R11022314880	C	T	.	.	PR;ANN=T|upstream_gene_variant|MODIFIER|EPlOSAG00000037021|EPlOSAG0000037 21|transcript|EPlOSAT00000038409|lincRNA||n.-3625C>T|||||3625|, T|upstream_gene_variant|MODIFIER|EPlOSAG00000001220|EPlOSAG00000001220|transcript| EPlOSAT00000002608|lincRNA||n.-3647C>T|||||3647|, T|upstream_gene_variant|MODIFIER|OS11G0588400|OS11G0588400|transcript|OS11T0588400 02|protein_coding||c.-3236C>T|||||3236|WARNING_TRANSCRIPT_NO_START_CODON, T|downstream_gene_variant|MODIFIER|OS11G0588300|OS11G0588300|transcript|OS11T05883 00-01|protein_coding||c.*5082C>T|||||4799|, T|downstream_gene_variant|MODIFIER|EPlOSAG00000034867|EPlOSAG00000034867|transcrip t|EPlOSAT00000036255|lincRNA||n.*3625G>A|||||3625|, T|downstream_gene_variant|MODIFIER|OS11G0588300|OS11G0588300|transcript|OS11T05883 00-02|protein_coding||c.*5082C>T|||||4799|, T|intron_variant|MODIFIER|OS11G0588400|OS11G0588400|transcript|OS11T0588400-01|protein_coding|2/4|c.-6+147C>T||||||
**LOC_Os11g37740**	22315943	R11022315943	A	T	.	.	PR;ANN=T|synonymous_variant|LOW|OS11G0588400|OS11G0588400|transcript|OS11T05 88400-01|protein_coding|3/5|c.687A>T|p.Leu229Leu|964/3330|687/2736|229/911||, T|upstream_gene_variant|MODIFIER|EPlOSAG00000037021|EPlOSAG00000037021|transcript| EPlOSAT00000038409|lincRNA||n.-2562A>T|||||2562|, T|upstream_gene_variant|MODIFIER|EPlOSAG00000001220|EPlOSAG00000001220|transcript| EPlOSAT00000002608|lincRNA||n.-2584A>T|||||2584|, T|upstream_gene_variant|MODIFIER|OS11G0588400|OS11G0588400|transcript|OS11T0588400 02|protein_coding||c.-2173A>T|||||2173|WARNING_TRANSCRIPT_NO_START_CODON, T|downstream_gene_variant|MODIFIER|EPlOSAG00000034867|EPlOSAG00000034867|transcrip t|EPlOSAT00000036255|lincRNA||n.*2562T>A|||||2562|
**LOC_Os11g37740**	22316950	R11022316950	C	T	.	.	PR;ANN=T|missense_variant|<u>**MODERATE**</u>|OS11G0588400|OS11G0588400|transcript|OS1 1T0588400-01|protein_coding|4/5|c.1205C>T|p.Thr402Ile|1482/3330|1205/2736|402/911||, T|upstream_gene_variant|MODIFIER|EPlOSAG00000037021|EPlOSAG00000037021|transcript| EPlOSAT00000038409|lincRNA||n.-1555C>T|||||1555|, T|upstream_gene_variant|MODIFIER|EPlOSAG00000001220|EPlOSAG00000001220|transcript| EPlOSAT00000002608|lincRNA||n.-1577C>T|||||1577|, T|upstream_gene_variant|MODIFIER|OS11G0588400|OS11G0588400|transcript|OS11T0588400 02|protein_coding||c.-1166C>T|||||1166|WARNING_TRANSCRIPT_NO_START_CODON, T|downstream_gene_variant|MODIFIER|EPlOSAG00000034867|EPlOSAG00000034867|transcrip t|EPlOSAT00000036255|lincRNA||n.*1555G>A|||||1555|
**LOC_Os11g37740**	22318020	R11022318020	C	T	.	.	PR;ANN=T|missense_variant|<u>**MODERATE**</u>|OS11G0588400|OS11G0588400|transcript|OS1 1T0588400-01|protein_coding|4/5|c.2275C>T|p.Arg759Trp|2552/3330|2275/2736|759/911||, T|upstream_gene_variant|MODIFIER|EPlOSAG00000037021|EPlOSAG00000037021|transcript| EPlOSAT00000038409|lincRNA||n.-485C>T|||||485|, T|upstream_gene_variant|MODIFIER|EPlOSAG00000001220|EPlOSAG00000001220|transcript| EPlOSAT00000002608|lincRNA||n.-507C>T|||||507|, T|upstream_gene_variant|MODIFIER|OS11G0588400|OS11G0588400|transcript|OS11T0588400 02|protein_coding||c.-96C>T|||||96|WARNING_TRANSCRIPT_NO_START_CODON, T|downstream_gene_variant|MODIFIER|EPlOSAG00000034867|EPlOSAG00000034867|transcrip t|EPlOSAT00000036255|lincRNA||n.*485G>A|||||485|
**LOC_Os11g37860**	**22432467***	R11022432467	G	A	.	.	PR;ANN=A|missense_variant|<u>**MODERATE**</u>|OS11G0590700|OS11G0590700|transcript|OS 1T0590700-01|protein_coding|3/6|c.931G>A|p.Glu311Lys|1237/3566|931/2565|311/854||
**LOC_Os11g37870**	22443270	R11022443270	C	T	.	.	PR;ANN=T|upstream_gene_variant|MODIFIER|OS11G0590900|OS11G0590900|transcript OS11T0590900-00|protein_coding||c.-858C>T|||||858|, T|intergenic_region|MODIFIER|OS11G0590700-OS11G0590900|OS11G0590700-OS11G0590900|intergenic_region|OS11G0590700-OS11G0590900|||n.22443270C>T||||||

<table>
<tr><td>LOC_Os11g37870</td><td>22444386*</td><td>R11022444386</td><td>G</td><td>C</td><td>.</td><td>.</td></tr>
<tr><td colspan="7">PR;ANN=C|missense_variant|MODERATE|OS11G0590900|OS11G0590900|transcript|OS
1T0590900-00|protein_coding|1/1|c.259G>C|p.Glu87Gln|259/1266|259/1266|87/421||</td></tr>
<tr><td>LOC_Os11g37960</td><td>22507045*</td><td>R11022507045</td><td>A</td><td>G</td><td>.</td><td>.</td></tr>
<tr><td colspan="7">PR;ANN=G|synonymous_variant|LOW|OS11G0592100|OS11G0592100|transcript|OS11T0
592100-01|protein_coding|2/2|c.300A>G|p.Thr100Thr|363/730|300/459|100/152||,
G|upstream_gene_variant|MODIFIER|OS11G0592200|OS11G0592200|transcript|OS11T0592200
01|protein_coding||c.-4681A>G|||||4461|,
G|downstream_gene_variant|MODIFIER|OS11G0592000|OS11G0592000|transcript|OS11T0592
00-01|protein_coding||c.*4342A>G|||||4122|</td></tr>
<tr><td>LOC_Os11g38870</td><td>23132523</td><td>R11023132523</td><td>C</td><td>T</td><td>.</td><td>.</td></tr>
<tr><td colspan="7">PR;ANN=T|missense_variant|MODERATE|OS11G0601600|OS11G0601600|transcript|OS
1T0601600-01|protein_coding|6/6|c.1874C>T|p.Ala625Val|2048/2306|1874/1959|625/652||,
T|upstream_gene_variant|MODIFIER|OS11G0601650|OS11G0601650|transcript|OS11T0601650
00|protein_coding||c.-1763G>A|||||39|WARNING_TRANSCRIPT_INCOMPLETE,
T|upstream_gene_variant|MODIFIER|EPlOSAG00000031993|EPlOSAG00000031993|transcript|
EPlOSAT00000033381|lincRNA||n.-1825G>A|||||1825|,
T|downstream_gene_variant|MODIFIER|OS11G0601700|OS11G0601700|transcript|OS11T0601
00-01|protein_coding||c.*714G>A|||||289|,
T|downstream_gene_variant|MODIFIER|EPlOSAG00000025115|EPlOSAG00000025115|transcri
t|EPlOSAT00000026503|lincRNA||n.*1945G>A|||||1945|</td></tr>
<tr><td>LOC_Os11g38870</td><td>23134862</td><td>R11023134862</td><td>C</td><td>T</td><td>.</td><td>.</td></tr>
<tr><td colspan="7">PR;ANN=T|upstream_gene_variant|MODIFIER|OS11G0601650|OS11G0601650|transcript
OS11T0601650-00|protein_coding||c.-
4102G>A|||||2378|WARNING_TRANSCRIPT_INCOMPLETE,
T|upstream_gene_variant|MODIFIER|EPlOSAG00000031993|EPlOSAG00000031993|transcript|
EPlOSAT00000033381|lincRNA||n.-4164G>A|||||4164|,
T|upstream_gene_variant|MODIFIER|EPlOSAG00000025115|EPlOSAG00000025115|transcript|
EPlOSAT00000026503|lincRNA||n.-
288G>A|||||288|,T|downstream_gene_variant|MODIFIER|OS11G0601600|OS11G0601600|tran
cript|OS11T0601600-01|protein_coding||c.*2254C>T|||||2081|,
T|intron_variant|MODIFIER|OS11G0601700|OS11G0601700|transcript|OS11T0601700-
01|protein_coding|3/4|c.220+413G>A||||||</td></tr>
<tr><td>LOC_Os11g38870</td><td>23135943</td><td>R11023135943</td><td>G</td><td>T</td><td>.</td><td>.</td></tr>
<tr><td colspan="7">PR;ANN=T|upstream_gene_variant|MODIFIER|OS11G0601650|OS11G0601650|transcript
OS11T0601650-00|protein_coding||c.-
5183C>A|||||3459|WARNING_TRANSCRIPT_INCOMPLETE,
T|upstream_gene_variant|MODIFIER|EPlOSAG00000025115|EPlOSAG00000025115|transcript|
EPlOSAT00000026503|lincRNA||n.-1369C>A|||||1369|,
T|downstream_gene_variant|MODIFIER|OS11G0601600|OS11G0601600|transcript|OS11T0601
00-01|protein_coding||c.*3335G>T|||||3162|,
T|intron_variant|MODIFIER|OS11G0601700|OS11G0601700|transcript|OS11T0601700-
01|protein_coding|2/4|c.105-553C>A||||||</td></tr>
<tr><td>LOC_Os11g38870</td><td>23136993*</td><td>R11023136993</td><td>T</td><td>A</td><td>.</td><td>.</td></tr>
<tr><td colspan="7">PR;ANN=A|upstream_gene_variant|MODIFIER|OS11G0601650|OS11G0601650|transcript
OS11T0601650-00|protein_coding||c.-
6233A>T|||||4509|WARNING_TRANSCRIPT_INCOMPLETE,
A|upstream_gene_variant|MODIFIER|EPlOSAG00000025115|EPlOSAG00000025115|transcript
EPlOSAT00000026503|lincRNA||n.-2419A>T|||||2419|
A|downstream_gene_variant|MODIFIER|OS11G0601600|OS11G0601600|transcript|OS11T0601
00-01|protein_coding||c.*4385T>A|||||4212|,
A|intron_variant|MODIFIER|OS11G0601700|OS11G0601700|transcript|OS11T0601700-
01|protein_coding|1/4|c.15-70A>T||||||</td></tr>
<tr><td>LOC_Os11g40840</td><td>24437193</td><td>R11024437193</td><td>A</td><td>G</td><td>.</td><td>.</td></tr>
<tr><td colspan="7">PR;ANN=G|upstream_gene_variant|MODIFIER|EPlOSAG00000003843|EPlOSAG0000000
843|transcript|EPlOSAT00000005231|lincRNA||n.-4709A>G|||||4709|,</td></tr>
</table>

							G|intergenic_region|MODIFIER|OS11G0624400-EPlOSAG00000003843|OS11G0624400-EPlOSAG00000003843|intergenic_region|OS11G0624400-EPlOSAG00000003843|||n.24437193A>G||||||
**LOC_Os11g40840**	**24438215***	R11024438215	T	A	.	.	PR;ANN=A|upstream_gene_variant|MODIFIER|EPlOSAG00000003843|EPlOSAG00000003843|transcript|EPlOSAT00000005231|lincRNA||n.-3687T>A|||||3687|, A|intergenic_region|MODIFIER|OS11G0624400-EPlOSAG00000003843|OS11G0624400-EPlOSAG00000003843|intergenic_region|OS11G0624400-EPlOSAG00000003843|||n.24438215T>A||||||
**LOC_Os11g45620**	**27605223***	R11027605223	T	C	.	.	PR; C|missense_variant|**<u>MODERATE</u>**|OS11G0682600|OS11G0682600|transcript|OS11T0682600-01|protein_coding|1/1|c.565A>G|p.Arg189Gly|3921/4045|565/689|189/228||WARNING_TRANS CRIPT_INCOMPLETE C|upstream_gene_variant|MODIFIER|OS11G0682501|OS11G0682501|transcript|OS11T0682501 00|protein_coding||c.-731A>G|||||731| C|downstream_gene_variant|MODIFIER|EPlOSAG00000007455|EPlOSAG00000007455|transcri pt|EPlOSAT00000008843|lincRNA||n.*890T>C|||||890|
